# A decagon wave around Saturn’s south pole

**DOI:** 10.1126/sciadv.aee4251

**Published:** 2026-09-02

**Authors:** Agustín Sánchez-Lavega, Amy A. Simon, Michael H. Wong, Leigh N. Fletcher, Arrate Antuñano, Ricardo Hueso, Peio Iñurrigarro, Aida Flix-Bellmunt, Arnau Miró, Enrique García-Melendo, Trevor Barry, Jean-Paul Oger, Glenn S. Orton, Itziar Garate-Lopez

**Affiliations:** ^1^ Escuela de Ingeniería de Bilbao, Universidad del País Vasco EHU, Bilbao 48013, Spain.; ^2^ NASA Goddard Space Flight Center/690, Greenbelt, MD 20771, USA.; ^3^ University of California, Berkeley, CA 94720, USA.; ^4^ School of Physics & Astronomy, University of Leicester, Leicester, UK.; ^5^ Escola Superior d’Enginyeries Industrial, Aeroespacial i Audiovisual de Terrassa, Universitat Politècnica de Catalunya, Terrassa (Barcelona) 08222, Spain.; ^6^ Broken Hill Observatory, Broken Hill, Australia.; ^7^ Association Française d’Astronomie, 75014 Paris, France.; ^8^ Jet Propulsion Laboratory, California Institute of Technology, Pasadena, CA 91109, USA.

## Abstract

Saturn has a unique long-lived, nearly stationary hexagon wave close to its north pole that is coupled to an intense eastward jet. Despite decades of observation, no similar wave has been reported so far in the southern hemisphere or elsewhere on Saturn. Ground-based and Hubble Space Telescope (HST) images obtained in 2025 revealed the presence of a decagonal wave at planetographic latitudes ∼58°S to 63°S. HST images in 2023 and 2024 showed vertices with weaker albedo contrasts and linear sides of the decagon, implying an evolving phenomenon. The decagon moves eastward with a velocity of 2.5 meters per second, much slower than the zonal jet at 60.5°S with 116 meters per second, and the longitudes of its vertices oscillate with a period of 32 days and amplitudes of 4.6° to 8.4°. From its properties, the decagon could be a large-scale meandering wave trapped vertically and confined meridionally by the dominant jet curvature. Shallow water model simulations suggest that the decagon may have originated from a spatially periodic disturbance in the jet peak or may have been driven by a dark anticyclonic vortex immediately to the north.

## INTRODUCTION

**Fig. 1.**
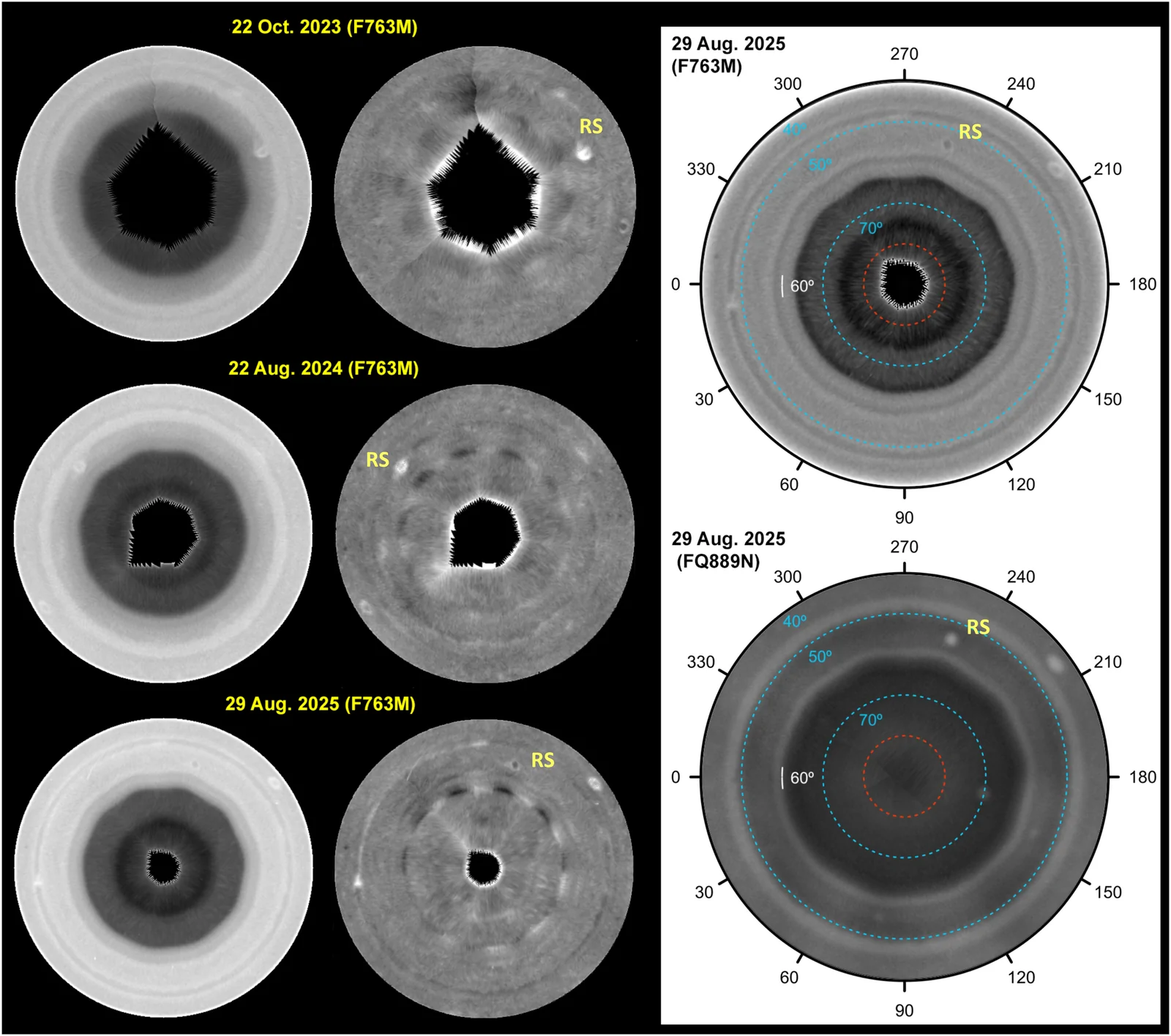
Polar maps of Saturn showing the decagon wave. Polar map projections of Saturn from 40°S to 90°S from HST images showing the temporal evolution of the decagon at two wavelengths. The two left columns show the maps over time (first column) and maps generated by subtracting the longitudinal mean brightness (second column) showing the decagon troughs (white spots) and crests (vertices appearing as dark spots). A Minnaert function and an unsharp mask were applied to correct the limb darkening and enhance the contrast, respectively. The right column shows the decagon at red continuum wavelengths sounding the deeper clouds (763 nm, upper map) and in the absorption methane band at 889 nm sounding upper tropospheric aerosols (bottom map). Latitude 60°S is identified by a white bar. Blue dashed lines mark the circles of latitude (red marks 80°S).

Saturn’s north pole hosts a unique atmospheric feature in the Solar System: a persistent hexagonal wave centered near the 78.5°N planetographic latitude (*1*, *2*). The hexagon was found in images obtained during the Voyager flybys in 1980–1981 and has been observed with ground-based telescopes and Hubble Space Telescope (HST) and by the Cassini spacecraft since then, indicating a lifetime that exceeds 44 years (*3*–*7*). The hexagon also manifests in the temperature field, extending its influence above the upper clouds into the stratosphere (*8*, *9*). The hexagon is a stable atmospheric wave that sits on an eastward jet with a peak speed of ∼100 ms^−1^ while remaining nearly stationary in longitude with respect to Saturn’s radio-rotation System III (*10*–*12*). Because of the long-term stability of Saturn’s zonal jet system (*13*–*15*), it could be expected that an analog wave could form in one of the two subpolar jets in the southern hemisphere at 60°S and 73.6°S. However, no such wave has been previously reported (*16*), although an ephemeral partial polygonal feature was observed in 2004 by Cassini spacecraft (*17*). Because of Saturn’s axial tilt, the visibility of its southern hemisphere from Earth ended in mid-2012, and it was not until 2023 that it became visible again (fig. S1). Despite the low viewing angle of the southern hemisphere, in October 2023, the HST red-filtered images (wavelength >600 nm) showed the presence of a 10-sided polygonal structure—a decagon at planetographic latitude ∼63°S (Fig. 1). In August 2024, with improving viewing angle, the decagon sides and vertices became more visible, although their contrast was not uniform in longitude (Fig. 1).

## RESULTS

HST images in August and September 2025 show the 10 vertices of the decagon fully developed, although with different contrasts across the latitude circle (Fig. 1). Three vertices (and sides) were very prominent, four were less prominent, and three were less defined. Ground-based images from 2025 also captured the decagon (fig. S2). The latitude of the wave center in the red filters at wavelengths of 631 nm, 727 nm (a weak methane absorption band), 763 nm, and 937 nm was 63°S ± 0.4°. The wave appeared displaced equatorward in the 889-nm strong methane band, centered at 60.5°S ± 0.4° (Fig. 1). The decagon wave (wave number *n* = 10) has a mean zonal wavelength *L_x_* = 16,782 ± 1100 km, and the edges of the crests and troughs define a sinusoidal wave with a latitudinal peak-to-peak amplitude of 2.7° ± 0.3° (2645 km).

A similar but less defined decagonal feature is observed outside the main decagon at 763 nm in a dark belt centered at 53°S in 2023 and 2024 and at 58°S in 2025 (Fig. 1). In 2025, the decagonal undulation is also observable as a white band in HST images in the methane absorption band at 889 nm at 58.8°S (Fig. 1). The latitudinal width of these bands is ∼2° to 3° (∼2000 to 3000 km). This banding and the meridional reflectivity profiles at different wavelengths (fig. S3) reveal that the decagon patterns extend from latitudes ∼53°S to ∼66°S (∼13,000 km), suggesting a vertically layered, multilevel phenomenon extending over a broad pressure range, from at least 10 mbar to 2 bar (tables S1 and S2), i.e., similar to the hexagon (*18*, *19*).

The decagon pattern is close to a Red Spot (RS) centered at 55°S and observed from 2023 to 2025 (Fig. 1 and fig. S4). The RS has a size of about 4000 km and was well tracked in ground-based images from 2023 to 2025, moving with a velocity of 23 ± 1 ms^−1^. At 763 nm, the RS showed a high albedo in 2023 and 2024 but appeared darker in 2025. The RS was bright at 889 nm but dark in the ultraviolet from 2023 to 2025 (fig. S4). According to the wind profile (*15*), the RS is an anticyclone, which is consistent with its brightness at 889 nm (i.e., had higher cloud tops relative to background clouds; table S2). The RS is similar to the spot observed in 1981 by Voyager 1 and Voyager 2 that was informally nicknamed as “Anne’s spot” (*13*, *20*). The role of the RS in the formation of the decagon remains unclear, as was initially the case between Anne’s spot and the hexagon (*13*, *21*, *22*). We see the highest vertex contrast near RS, and the section of the decagon with indistinct crests of the wave is on the opposite hemisphere from the RS (Fig. 1).

### Longitude motions and wind velocity

**Fig. 2.**
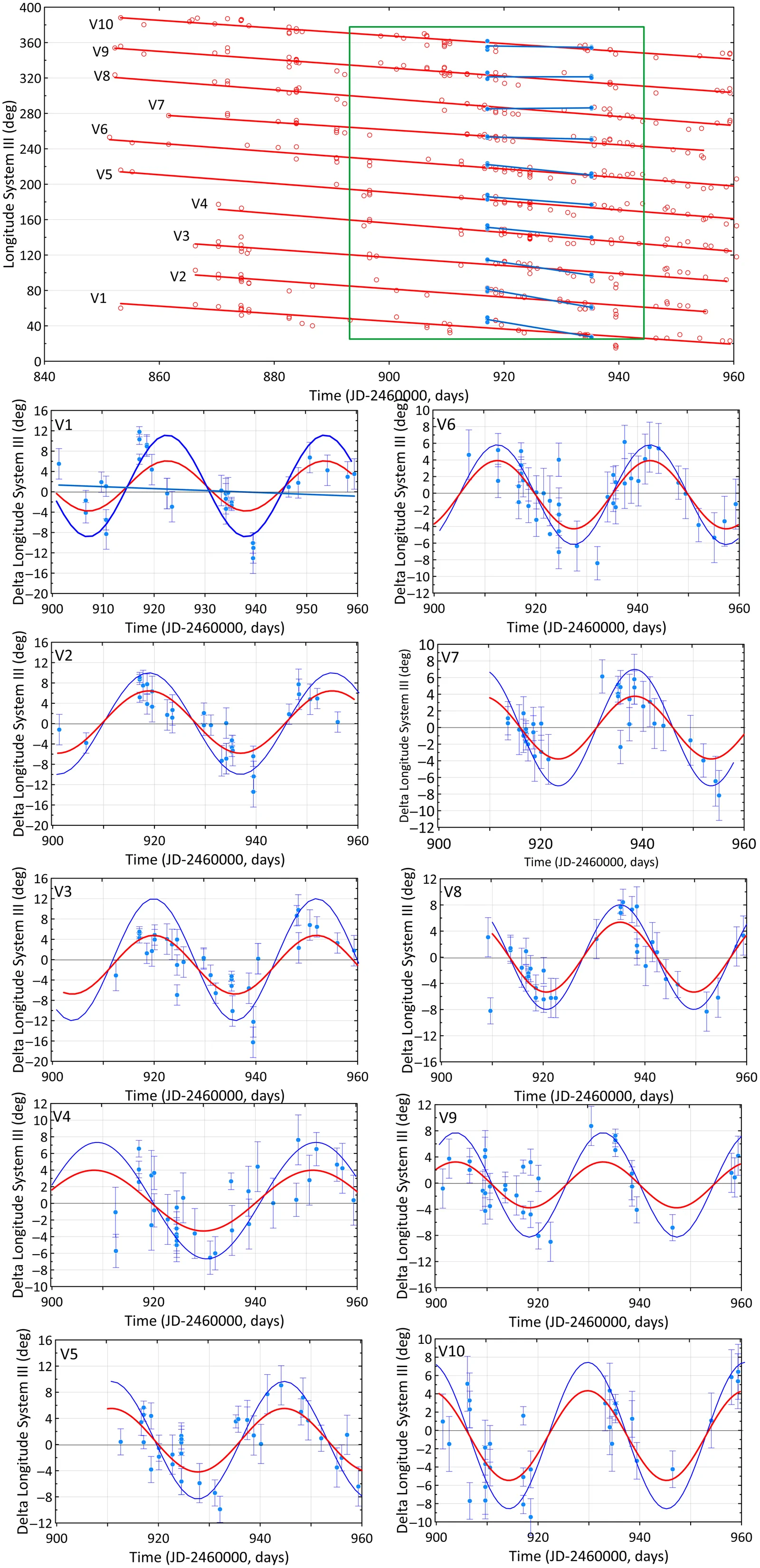
Motions in longitude of the decagon vertices. The vertices are identified as V1 to V10. The upper panel shows their longitudinal drift in System III and the linear fit to the motion (red circles and line, respectively). The HST data are identified by blue dots and by the blue line (a linear fit). The green box in the top panel delimits the dataset used to study the zonal oscillations. The lower set of panels show the detrended oscillating amplitude from the linear fit for each vertex and the sinusoidal regression curve (red) with the blue curve showing the upper limit of the amplitude oscillation.

The decagon motion was measured by tracking the position in longitude of its vertices in red images (wavelength range of ∼650 to 950 nm) obtained with HST and ground-based telescopes from the ALPO Japan and Planetary Virtual Observatory Laboratory (PVOL) databases [(*23*); see Data, code, and materials availability]. The period covered extends from 26 June to 11 October 2025. We use the System III reference frame for longitude measurements (rotation rate = 810.7939024°/day and rotation period = 10 hours, 39 min, and 22.4 s) (*24*) and planetographic latitudes. A linear fit to the mean drift in longitude of the 10 vertices gives a value of −0.47 ± 0.035°/day, the negative sign indicating eastward motion relative to System III (Fig. 2, upper panel). This corresponds to an absolute rotation period for the decagon of 10 hours, 39 min, and 1.8 ± 1.6 s and a mean zonal velocity at latitude 63°S of *u*_DEC_ = +2.5 ± 0.2 ms^−1^.

The zonal drift of each vertex oscillates relative to the linear fit, as shown in Fig. 2 (lower panel). We have studied these oscillations in the observation time period with the best data including HST measurements and covering more than a full oscillation. The detrended amplitude, i.e., the differences in longitude for each vertex relative to the linear fit of its motion, has been fitted to a sinusoidal curve of the form $A=A_{0}\;\text{sin}\left[\left(2\pi t/\tau \right)+\varphi_{0}\right]$, where *A* is the amplitude, τ is the period, *t* is the time, and φ_0_ is a phase shift. The resulting longitudinal oscillation of the vertices has a mean period τ = 32.5 days (with only one of the vertices deviating from this value with a period of 43 days) and a mean amplitude *A*_0_ = 4.6° with a maximum value *A*_0_ (max) = 8.4° (table S3). This kind of longitudinal oscillation is similar to that observed in Jupiter’s Great Red Spot that has a period of ∼90 days and an amplitude of ∼1° (*25*–*27*). Furthermore, the date on which the peak of the oscillation occurs (for example, the date of the minima in Fig. 2) is shifted between two consecutive vertices by an average of 2.7 days (fig. S5). These properties in the movement of the decagon indicate the existence of a complex dynamical behavior, never observed in the hexagon, becoming a subject that deserves future attention.

**Fig. 3.**
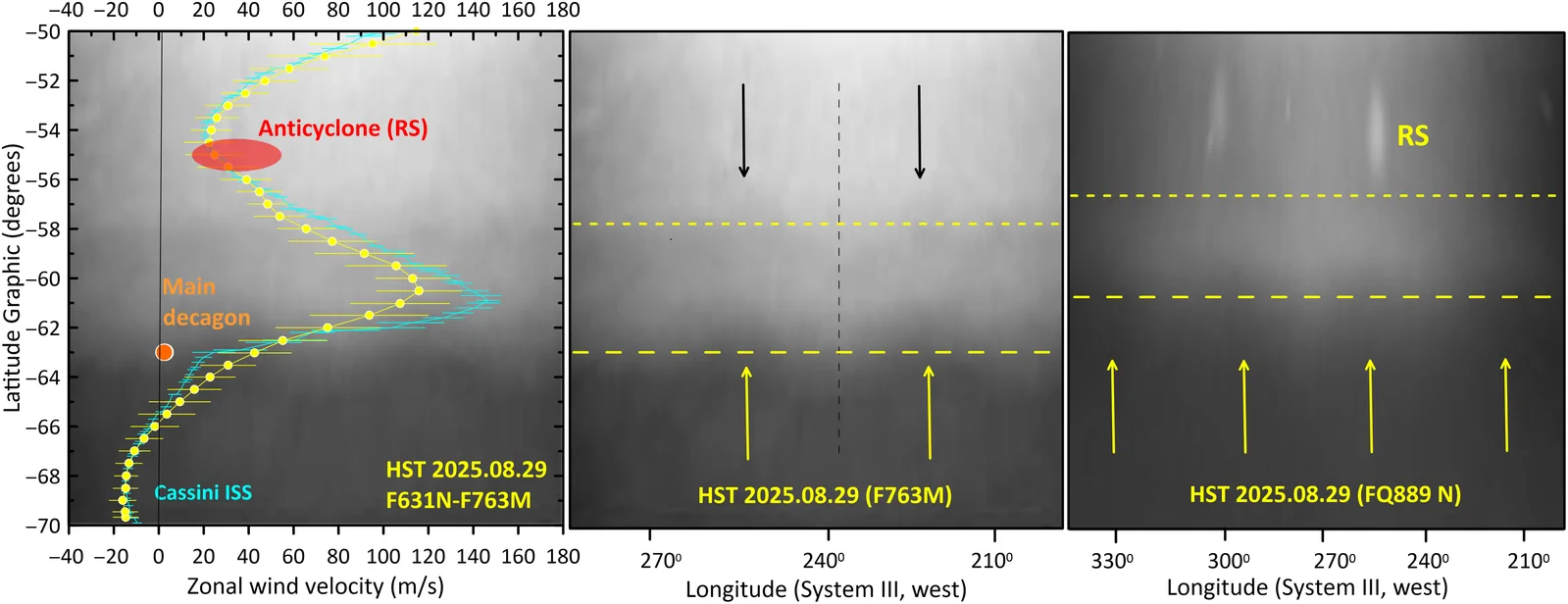
Zonal winds and maps of the decagon. Left panel: Saturn’s zonal wind profile from 50°S to 70°S measured in 2025 using HST image pairs (yellow dots) compared to that derived from Cassini (light blue line) (*15*). The orange dot marks the velocity of the decagon vertices as measured at 763 nm. The red oval marks the velocity of the RS (not to scale). Central panel: Part of the decagon at 763 nm with two vertices identified by yellow arrows (this map constitutes the background shown in the left panel). The vertices in the northern dark belt at 58°S have much lower contrasts and are identified by black arrows. Right panel: Cylindrical projection of an FQ889N image showing four decagon vertices (identified by yellow arrows) and RS. Longitude scales vary in all panels to show more than one decagon vertex, making RS distorted in the right panel. Note also the different latitudes of the vertices with wavelength (central and right panels) identified by the dotted and dashed yellow lines.

We have used HST images from August 2025 to measure the zonal wind velocity profile *u*(φ) shown in Fig. 3. The vertices of the main decagon as observed in filters F763M and FQ889N are located in the poleward side of a jet that has a peak centered at 60.5°S with a speed *u*_0_ = 116 ms^−1^. The shape and strength of this jet have not changed substantially since 1981, as also observed in the other nonequatorial jets, within measurements errors (*13*–*15*). The decagon moves with a velocity relative to the jet at its latitude of *u*_DEC_ − *u*(63°S) = −35 ms^−1^ (filter F763M). However, as mentioned above, the decagon morphology is also observed in bands located further north, occupying the entire latitude range over which the jet extends (cyclonic and anticyclonic sides) (Figs. 1 and 3 and fig. S6). For this reason, we should consider the velocity of the decagon relative to the peak of the jet and then *u*_DEC_ − *u*_0_ ∼ −113 ms^−1^. The negative sign indicates westward motion relative to the zonal jet. The dynamical situation is analogous to that observed in the northern hexagon (*1*–*7*).

### Temperature and thermal winds

Infrared images of Saturn were obtained on 16 and 17 August 2025 using the VLT (Very Large Telescope) Imager and Spectrometer for mid-InfraRed (VISIR) mounted on the 8.2-m-diameter telescope UT-2 unit of the European Southern Observatory (ESO) (*28*, *29*). The images cover the spectral range from 7.7 to 19.5 μm and provide brightness temperatures equivalent to radiances that sound the upper troposphere (100 to 300 mbar) and stratosphere (1 to 5 mbar) (fig. S7) (*30*). Brightness temperature map projections of the south polar region do not show a clear signature of the decagon vertices because of the high viewing angle of the south polar region (fig. S8). Instead, the images show zonal banding, in particular in the wavelength range of 17.6 to 19.5 μm sounding the upper troposphere (*29*), with one of these brightness temperature bands corresponding to the decagon region (fig. S9).

**Fig. 4.**
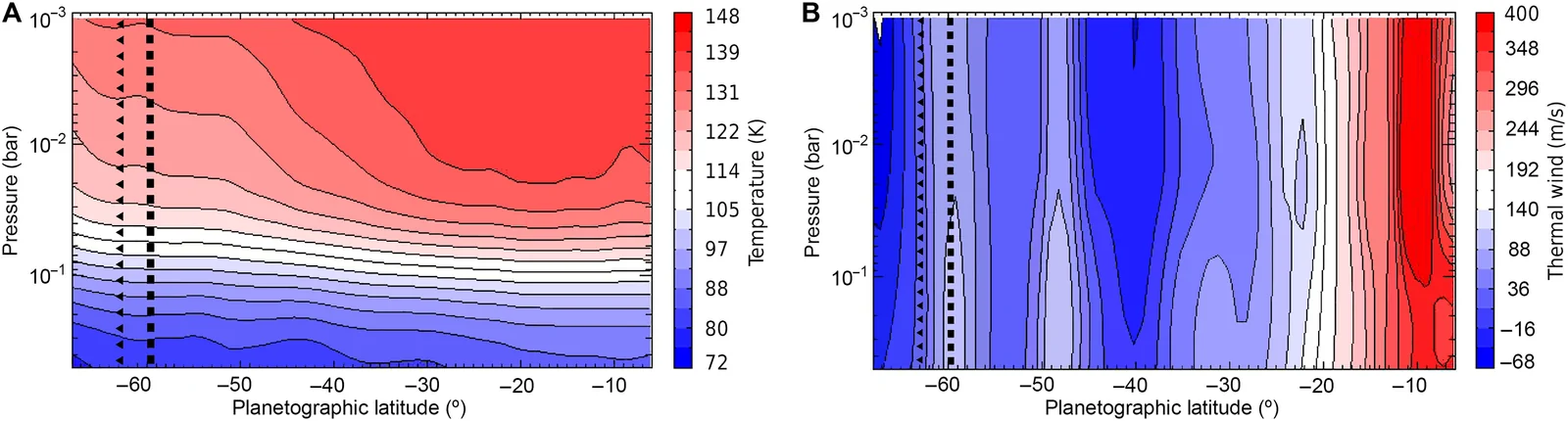
Temperature maps and thermal winds. (**A**) Equator to pole temperatures in Saturn’s southern hemisphere as a function of altitude (from 0.5 bar to 1 mbar) retrieved from VISIR thermal-IR observations in different spectral bands. (**B**) The vertical structure of the zonal wind velocity retrieved from the meridional temperature gradients and the thermal wind relation assumed that the cloud-tracked zonal flows are at the ∼500-mbar level. The vertical marks show the latitude of the wave vertices at wavelengths of 763 nm (triangles) and 889 nm (squares). Data are for 16 August 2025.

An estimate of the tropospheric and stratospheric temperatures was retrieved from VISIR imaging (Fig. 4A) (*30*). The temperature maps show a marked seasonal behavior, with cooler temperatures toward the springtime pole at the same pressure level and, superimposed, the temperature fluctuations related to the zonal jet structure. These temperature data are used to infer the vertical structure of the zonal jets from the meridional temperature gradient and the thermal wind balance equation (Fig. 4B) (*30*). The jet on which the decagon is located shows decreasing velocities with altitude (*31*), as do the two adjacent jets located equatorward (latitudes 48°S and 32°S). The wave is on the flanks of the prograde jet in a region away from the strongest negative vertical wind shear.

## DISCUSSION

The decagon is located in a zonal jet in geostrophic balance, given that the Rossby number is $\mathrm{Ro}=u_{0}/f_{0}L$ ∼ 0.04, for *u*_0_ = 116 ms^−1^, a Coriolis parameter $f_{0}=2\Omega_{\text{III}}\text{sin}\varphi_{0}$ = 2.7 × 10^−4^ s^−1^, and *L* = 2700 km from the jet width in latitude at half maximum ∼2.8°. The ambient vorticity ζ=∂u/∂y (i.e., the jet meridional wind shear) ranges from 2 × 10^−5^ s^−1^ in the anticyclonic side to −6 × 10^−5^ s^−1^ in the cyclonic side (∼0.1 to 0.5 times the planetary vorticity *f*_0_) (fig. S10). The ambient vorticity has an averaged peak curvature of |∂^2^*u*/∂*y*^2^| ∼ 2.5 × 10^−11^ m^−1^ s^−1^, which is approximately eight times the planetary vorticity gradient $\beta =2\Omega_{\text{III}}\text{cos}\varphi /R_{S}$ = 3.2 × 10^−12^ m^−1^ s^−1^ [*R*_S_ (φ) is Saturn’s radius at latitude φ; fig. S10]. With these data, and as proposed for the hexagon (*6*, *22*), the decagon could be a quasigeostrophic Rossby wave trapped vertically and confined meridionally by the dominant jet curvature [vertical wave number *m*^2^ < 0; (*6*)] and with an intrinsic phase speed *c_x_* − *u*_0_ ∼ −113 ms^−1^. However, the decagon is not uniform in longitude, unlike the hexagon whose sides and vertices are equally contrasted. The decagon did not exist in Saturn’s previous year, and it appears to be developing from 2023 to 2025, rather than the long-term steadiness of the unchanging hexagon over the insolation cycle (fig. S1). This would suggest that the decagon is a wave resulting from a dynamical instability of the jet occurring in the upper weather layer, as it has also been proposed for the hexagon (*32*–*36*). If that were the case, the decagon could be a transient evolving phenomenon, whereas the hexagon is a robust wave, as shown by its long lifetime and stability, even in the presence of nearby intense convective storm activity (*10*, *12*).

### Shallow water model simulations

**Fig. 5.**
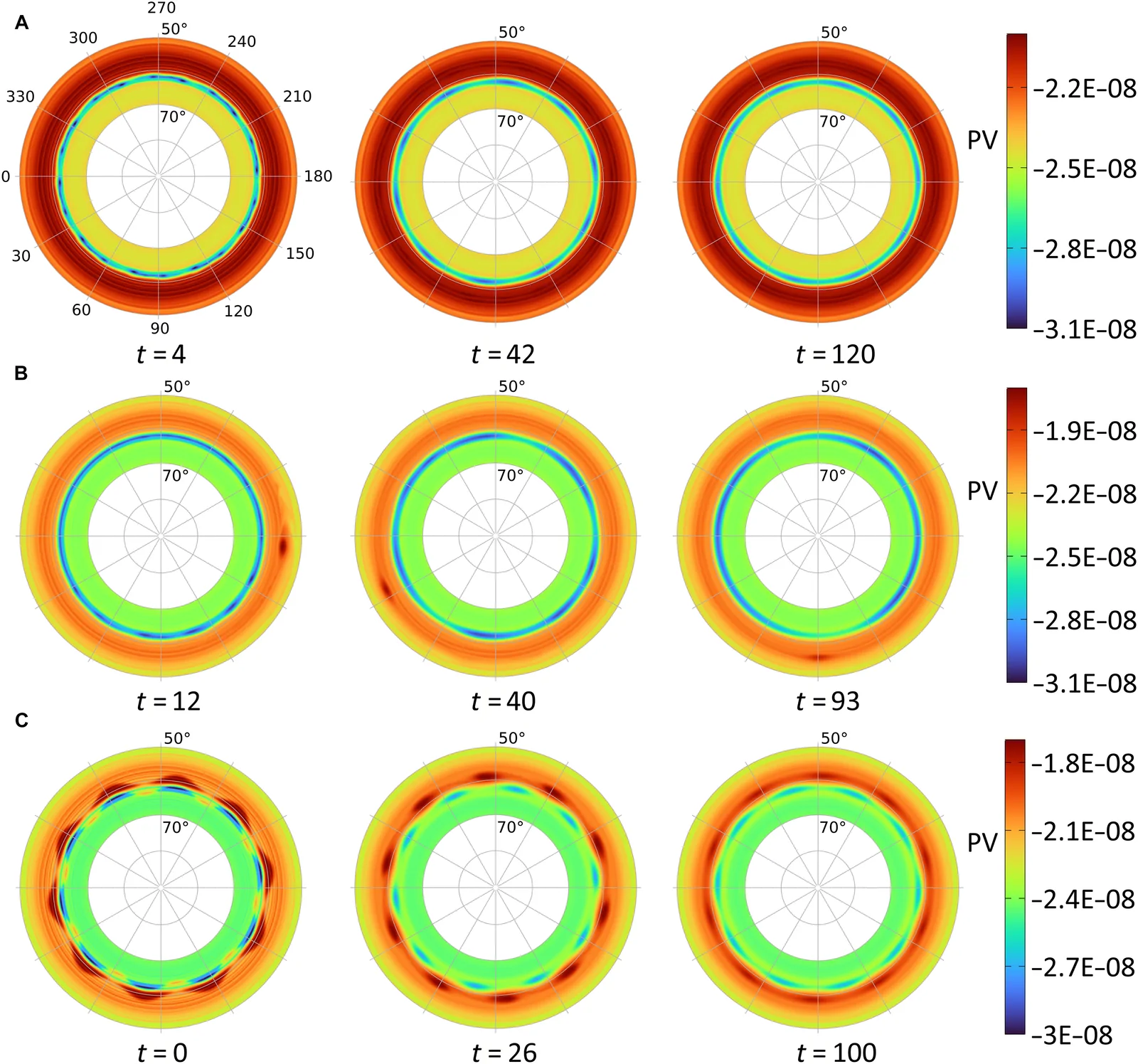
SW model results. (**A**) Map showing the evolution of the PV generated by an initial periodic disturbance with a wave number of 10 centered on the jet peak at 60.5°S. (**B**) Map showing the evolution of the PV perturbation caused by the presence of the RS anticyclone at 55°S with velocity at periphery *V*_t_ = 125 ms^−1^. (**C**) Map of the evolution of the PV when a periodic sinusoidal wave with an amplitude of 1.3° and a wave number of 10 is initially introduced on the jet peak. For all the cases, we use *H* = 12.5 km and *L*_D_ = 1329 km. Cylindrical map projections of these simulations are presented as fig. S11.

We have used a shallow water (SW) layer model (*10*, *12*, *37*, *38*) to perform a zero-order exploration of the formation and stability of the decagon wave. The SW represents a simplified version of Saturn’s troposphere at the latitude of the decagon characterized by a fluid depth *H* (we use a range of 5 to 50 km) that defines the Rossby deformation radius as $L_{D}=\sqrt{\mathrm{gH}}/f_{0}$ (range of 850 to 2700 km). We impose a fluid motion over perturbed velocities that follows the measured wind velocity profile. The fluid is represented by its vorticity field PV(λ,φ)=(ζ+f)/H or by the deviation height η(λ,φ) from the fluid depth *H*. We have run simulations for three cases: (i) evolution of the fluid following localized Gaussian disturbances in the jet, (ii) evolution of the fluid when an anticyclone like RS is introduced at its observed latitude, and (iii) evolution of a sinusoidal wave, with a Gaussian meridional profile and a wave number of 10 imposed initially on the jet peak at 60.5°S. Figure 5 shows a selection of results from these simulations. In case (i), by introducing 10 localized Gaussian perturbations equidistant in length, we obtain a stable pattern in potential vorticity field (PV) and η, centered on the jet, with a wave number of 10 (Fig. 5A). However, its phase velocity is 88 ms^−1^, much higher than that of the decagon. Using a smaller number of perturbations, the system relaxes to a pattern with a wave number equal to the number of perturbations introduced. In case (ii) (Fig. 5B), depending on the value of *H* and tangential velocity *V*_t_ of the RS anticyclone, we get cases that perturb the jet and generate a periodic pattern in PV and η. We cannot therefore rule out the possibility that the formation of RS is ultimately the cause of the decagon, which may survive even after the disappearance of the anticyclone. In case (iii) (Fig. 5C), a sinusoidal wave in the PV field (wave number of 10) becomes rapidly unstable and fragments and forms a stable pattern of anticyclones (located on the northern side of the jet peak) and cyclones (on the southern side of the jet) with the same wave number. This pattern moves with a zonal speed of 46 ms^−1^, higher than that of the decagon but well below that of the jet peak. One possibility is that the decagon contains these vortices on either side of its undulation, even if they could not be observed because of the limited resolution of the images. Nevertheless, and even given the simplicity of the SW model, the generation of these periodic structures in the jet appears promising as a possible explanation for this phenomenon and encourages the conduct of simulations with more advanced multilayer models (*39*).

Saturn’s decagon offers a rare opportunity to probe polar jet dynamics and the processes that create stable polygonal flows in giant planets, including Jupiter’s polar cyclones arranged in polygonal patterns in a weak wind field beyond the 80° latitude (*40*). Models have replicated polygonal structures using various combinations of turbulence, jet instability, eddies and vortices, and waves in shallow and multilayer models that include vertical wind shear (*33**–**35*, *39*, *41**–**44*). The discovery and characterization of Saturn’s new decagon open the door to exploring conditions (e.g., wind profile structure and changing insolation) under which models can generate stable or unstable evolving polygonal structures.

## MATERIALS AND METHODS

### Image analysis and measurement procedure

We used images obtained in the visual range with the HST, Wide Field Camera 3 (WFC3), for the Outer Planets Atmospheric Legacy [OPAL; (*45*)] programs 16995, 17294, and 17843 on 22 October 2023, 22 August 2024, and 29 August 2025, respectively, and data from the Saturn Equinox program 18102 on 16 and 17 September 2025 (PI A. Simon). For OPAL programs, the following filters were used: F225W, F275W, F343N, F395N, F467M, F502N, F631N, F727N, F763M, and FQ889N. For the Saturn Equinox program 18102, filters used were as follows: F336N, F547M, F631N, F689M, FQ727N, FQ906N, and FQ937N. Details about the filters are available in (*46*, *47*). Each series of images covers two Saturn rotations.

We also used images obtained with the PlanetCam, a two-channel camera installed in the 2.2-m telescope at Calar Alto Observatory (*48*). Images were obtained on 29 August to 1 September 2025, with different filters in the visual channel (0.35 to 1 μm) and short-wave infrared (IR) channel (1 to 1.7 μm). Additional images were also provided by a network of observers around the world, contributing to the repositories at ALPO Japan and PVOL (Supplementary Materials). Most of these images were contrast enhanced with unsharp mask filters to improve the visibility of the decagon vertices and their measurement. We used WinJUPOS software to navigate the images and locate the positions of the decagon vertices and other features. The images were polar projected around the South Pole. Navigation errors in longitude and latitude location are ±0.1°. Because of the curvature at the vertices of the decagon, ground-based images have a precision in vertex longitudes of ±3° (for normal images), ±2° (for the best images), and ±1° for HST images.

### Zonal wind profile measurement

To derive the zonal wind profile, we used HST images from the OPAL program taken in August 2025. We selected all available pairs of images in the F631N and F763M filters, given that atmospheric features show the sharpest contrast, taken one Saturn rotation apart, so that the same features are visible but slightly displaced in longitude. These images were converted into equirectangular projection maps using the map computation tool of WinJUPOS and then high-pass filtered to enhance feature contrast.

Zonal velocities at each latitude were obtained using a one-dimensional brightness cross-correlation between pairs of maps (*49*). The latitudinal resolution of the maps is 0.1°/pixel, and correlations are computed for each row of pixels. This calculation yields the longitudinal shift (in degrees) corresponding to the maximum correlation value. Physically, this corresponds to the zonal displacement of features between the two cylindrical projections (*50*).

Limb darkening introduces a low-frequency signal in the brightness scans that can dominate the correlation results. Processing the images with a high-pass filter reduces the limb-darkening effect, although residuals remain depending on latitude. To further correct this, we applied a moving-average filter to the original scans and subtracted it from them, acting as an additional high-pass filter and producing flattened brightness profiles. The filter’s window size (i.e., the number of data points averaged) controls the performance of the filter, which should be short enough to remove the low-frequency limb darkening component but long enough to preserve the high-frequency variations that contain the actual information of the feature location. A shorter window, however, yields noisier measurements. Last, to obtain the zonal wind profile, the resulting data points were averaged in 0.5° latitude bins, smoothed over 2.5°, and the uncertainty was estimated as the standard deviation of the measurements within each range. Additional sources of error are described in (*15*).

### Brightness-temperature maps from VLT/VISIR

Saturn was observed through thermal-IR imaging by ESO’s VISIR (*30*) mounted on the 8.2-m UT2 telescope at Paranal as part of a long-term program of observations to track seasonal evolution of temperatures (*29*). Images were acquired in two 1-hour observing runs on consecutive nights (16 August 2025, 05:42 to 06:39 UT; 17 August 2025, 06:19 to 07:16 UT) as part of program 115.283U.001. Imaging conditions on both nights were excellent. Images were acquired in seven narrow-band filters between 7.9 and 19.5 μm, although one filter (8.6 μm) is omitted from analysis because of the low signal-to-noise ratio. The diffraction-limited angular resolution varies from 0.6″ at 19.5 μm to 0.25″ at 7.9 μm. Figure S7 shows images on 16 August 2025: Wavelengths between 17.6 and −19.5 μm sound temperatures in the upper troposphere (100 to 300 mbar) and reveal a belt/zone structure superimposed onto a seasonal gradient (from warm northern autumn to cool southern spring). Wavelengths at 7.9, 12.3, and 13.0 μm sound stratospheric methane, ethane, and acetylene, respectively, and can be used to provide a crude estimate of stratospheric temperatures.

Saturn is detected differentially on top of the bright sky background by rapid chopping and slower nodding between Saturn and the sky and then subtracting these beams to remove the sky emission and contributions from the telescope. Bad pixels are identified and cleaned before data are calibrated via scaling to a low-latitude average of Cassini/CIRS observations (*30*). Although Saturn’s temperatures are known to vary with time, the mean temperatures within ±30° of the equator are expected to remain approximately constant, and this crude radiometric calibration enables spectral radiances to be inverted to estimate atmospheric temperature. VISIR features a 1024 by 1024 Raytheon Aquarius IBC detector, with a pixel scale of 0.045″/pixel over a 38″ field of view, meaning that images were rotated to ensure that Saturn and its rings could fit within the field of view. A pattern of vertical and horizontal stripes was removed by Fourier-transforming the images into the spectral domain, identifying periodic structures to mask out, before reconstructing the image in the spatial domain (*29*). Last, we use PlanetMapper (*51*) to assign the geometry to each VISIR pixel, enabling polar projection (fig. S8) and extraction of the zonal mean brightness temperatures (fig. S9) for subsequent retrievals. The uncertainties shown in fig. S9 represent the average of the background sky observed in fig. S7.

The zonal-mean brightness in six filters (along with the estimate of uncertainty; fig. S9) was inverted to provide an estimate of the vertical temperatures as a function of latitude, from which temperature gradients can be extracted to estimate the vertical shear on the zonal winds. We use the NEMESIS spectral inversion code (*52*) to fit the six-point spectra at every latitude, presenting the results in Fig. 4 of the main text. The absolute temperatures at each pressure level are rather uncertain (with errors ranging from ∼2 K at 100 mbar to ∼4 K at 1 mbar), but the positions of the maxima in the temperature gradients are well constrained by VISIR imaging, revealing the vertical wind shear in the vicinity of the decagon. More precise constraints on the temperature field require thermal-IR spectroscopy.

### SW model description

An SW model is a simplified two-dimensional representation of the atmosphere. It consists of a thin layer of fluid on a spheroidal surface, and it is valid as long as the horizontal motion length scale (*L*) is significantly larger than the vertical scale (*H*). The SW code used in the simulations presented herein is described in (*37*, *53*) and has been used in prior work on Saturn (*10*, *12*, *38*). The model is built upon the following assumptions: The fluid is incompressible and inviscid, which decouples the dynamics from the thermodynamics. Given that *L* ≫ *H*, the system is assumed to be in hydrostatic equilibrium, and therefore, there are no vertical motions. The apparent gravity *g* is taken to be normal to the surface at every point, and also, only the normal component to the surface of the angular rotation vector is considered, for it is the one that contributes to the rotation effects. Zonal wind latitudinal variation is included in the simulations. These simplifications preserve the essential dynamics of planetary waves, geostrophic adjustment, and the hydrodynamic response to zonal winds. Consequently, the simulations account for the effects of rotation and the action of zonal jets in a planetary atmosphere. However, the model does not account for thermodynamics, stratification effects, and vertical motions. We use a single-layer model governed by the following equations

$$\frac{\mathrm{Du}}{\mathrm{Dt}}-\left(f+\frac{\text{sin}\varphi }{r_{z}}u\right)v=-\frac{g}{r_{z}}\frac{\partial \eta }{\partial \lambda }$$ (1)

$$\frac{\mathrm{Dv}}{\mathrm{Dt}}-\left(f+\frac{\text{sin}\varphi }{r_{z}}u\right)u=-\frac{g}{r_{m}}\frac{\partial \eta }{\partial \varphi }$$ (2)

$$\frac{\partial H}{\partial t}=-\nabla \cdot \left(h\;\vec{u}\right)+S\left(\lambda ,\varphi ,t\right)$$ (3)

where λ is the longitude; *h* is the fluid layer thickness; η is the fluid elevation above the reference level; *u* and *v* are the zonal and meridional velocities, respectively; *g = g*(φ) is the apparent gravity; and *S*(λ, φ, *t*) is any source (perturbation) of η, with other magnitudes defined above. The local zonal radius *r*_z_ and meridional radius *r*_m_ are defined as

$$r_{z}=\frac{R_{E}}{\sqrt{1+{\left(\frac{R_{p}}{R_{E}}\right)}^{2}{\text{tan}}^{2}\varphi }}$$ (4)

$$r_{m}=\frac{r_{z}/\text{cos}\varphi }{{\text{sin}}^{2}\varphi +{\left(\frac{R_{E}}{R_{p}}\right)}^{2}{\text{cos}}^{2}\varphi }$$ (5)

with *R*_E_ and *R*_P_ being the equatorial and polar radii of Saturn, respectively. Disturbances to the flow are produced by introducing variations of η with a Gaussian shape at a given time and location. A Gaussian surface elevation perturbation is equivalent to an injection of potential energy, which, under geostrophic adjustment, preserves its amplitude. The Gaussian disturbances are introduced as two-dimensional functions described by

$$\eta \;\left(\lambda ,\varphi \right)=h\text{exp}\left\{-{\left[{\left(\frac{\lambda -\lambda_{0}}{a}\right)}^{2}+{\left(\frac{\varphi -\varphi_{0}}{b}\right)}^{2}\right]}^{n}\right\}$$ (6)

where $a=\sqrt{2}\sigma_{\lambda },\;b=\sqrt{2}\sigma_{\varphi }$, *n* is a parameter that controls the flatness of the Gaussian top, and *h* is calculated given a characteristic velocity *V*_t_ imposing geostrophic equilibrium

$$h=\frac{r_{m}\;f\;b\;V_{t}}{2\;n\;g}{\left(1-\frac{1}{2n}\right)}^{\frac{2n-1}{2n}}\text{exp}\left(1-\frac{1}{2n}\right)$$ (7)

The case simulations started with an order zero run, without flow disturbances, to test the stability of the jet system along 200 days. Then, we proceed with the following cases: (i) series of 1 to 10 localized Gaussian disturbances equally separated in longitude, (ii) simulation of the RS anticyclone at 55°S to test its influence on the 61°S jet, and (iii) stability of a sinusoidal wave in the jet peak at 61°S with the wave number of 10, as observed for the decagon, to test its evolution. In tables S4 to S6, we give the range of values for the parameters tested in these simulations.

## Supplementary Materials

### This PDF file includes:

List of amateur contributors Figs. S1 to S11 Tables S1 to S6
