# Supplementary material for: A decagon wave around Saturn’s south pole
Source: Sci Adv. 2026 Sep 2;12(36):eaee4251. doi: 10.1126/sciadv.aee4251 (PMC13537229; doi:10.1126/sciadv.aee4251)
Supplement: Supplementary file 1 [file sciadv.aee4251_sm.pdf]

Science Advances
AAAS

Supplementary Materials for

A decagon wave around Saturn's south pole

Agustín Sánchez-Lavega *et al.*

Corresponding author: Agustín Sánchez-Lavega, agustin.sanchez@ehu.eus

Sci. Adv. **12**, eaee4251 (2026)
DOI: 10.1126/sciadv.aee4251

This PDF file includes:

List of amateur contributors
Figs. S1 to S11
Tables S1 to S6

List of Amateur Contributors

The following observers contributed with images to this study:

T. Akutsu (Japan), T. Barry (Australia), R. Cantemir (Germany), D. Capone (USA), P. Casquinha (Portugal), E. Chappel (USA), A. Cidadão (Portugal), J. L. Dauvergne (France), M. Duke (USA), C. Foster (South Africa), P. Gilet (France), C. Go (Philippines), V. González Cebrià (Spain), M. Hood (USA), S. Ito (Japan), T. Ishibashi (Japan), M. Kardasis (Greece), M. Karakas (Canada), Y. Kishimoto (Japan), C. Kolivas (Australia), T. Kumamori (Japan), M. Lewis (USA), V. Mirabella (Italy), I. Miyazaki (Japan), E. Morales (Puerto Rico), F. Morin (France), M. Morita (Japan), L. Morrone (Italy), T. Nishioka (Japan), J. P. Oger (France), C. Pellier (France), M. Phillips (USA), M. Portillo-León (Spain), R. Sedrani (Italy), C. Sprianu (Romania), E. Sussenbach (Curacao), M. Teodorescu (Romania), P. Tickner (UK), A. Vaccaro (Italy), J. Van der Meer (France), G. Walker (USA), A. Wesley (Australia), T. Williams (UK), M. Wölle (Austria)

Databases:

- Planetary Virtual Observatory Laboratory (PVOL)
  Image database: https://pvol2.ehu.eus/pvol2/https://pvol2.ehu.eus

- ALPO Japan, Association of Lunar and Planetary Observers in Japan
  Image database : https://alpo-j.sakura.ne.jp/indexE.htm

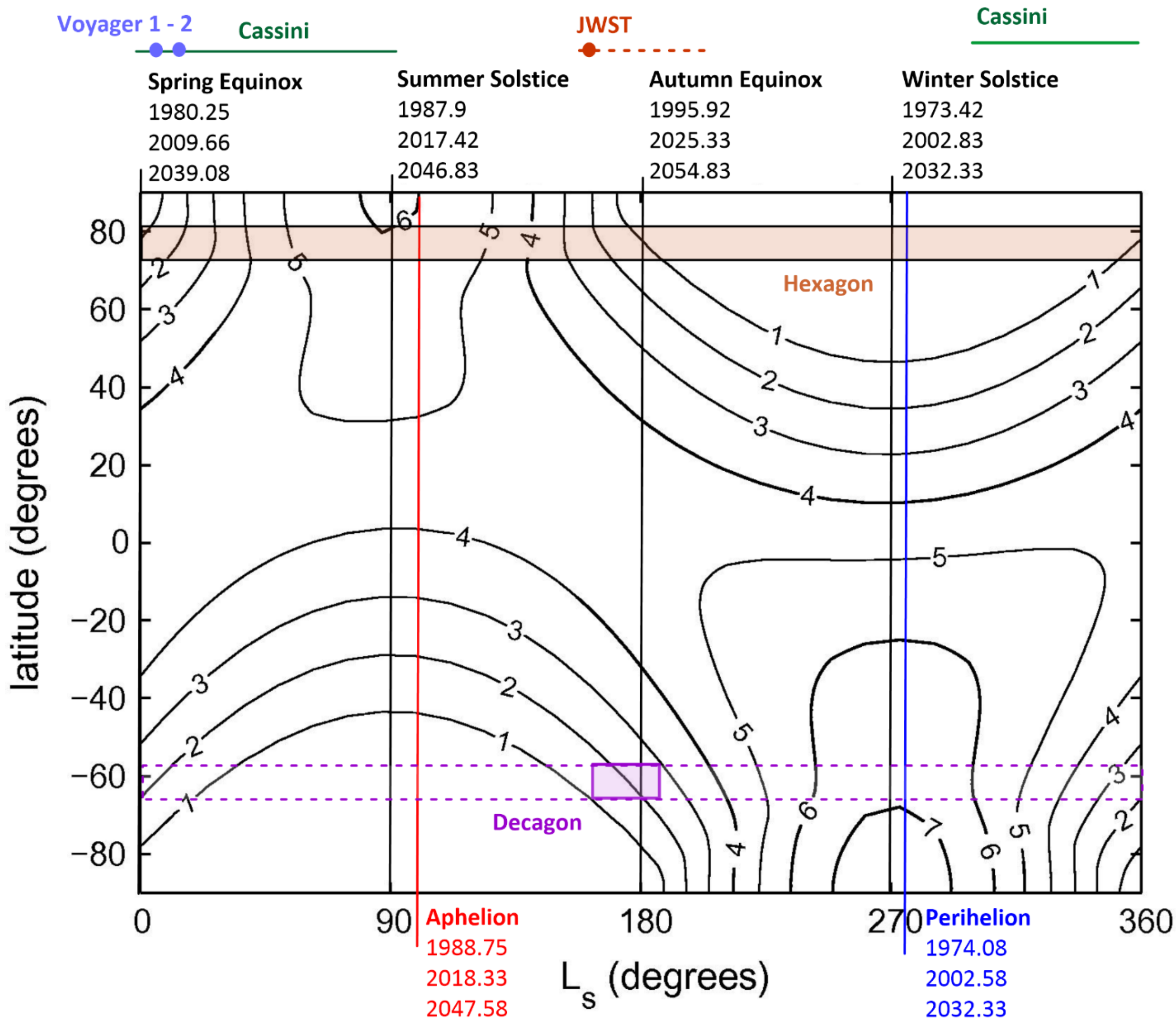

Figure S1. Insolation in Saturn's top of the atmosphere along the orbital period (in Wm^{-2}). In the upper and lower x-axis significant orbital epochs and dates are indicated. In the upper x-axis the different spacecraft observing periods are shown. The latitude and observing periods of the Hexagon and Decagon waves are also indicated. This plot does not consider the effects of the rings that do not affect the latitudes corresponding to the Hexagon and Decagon.

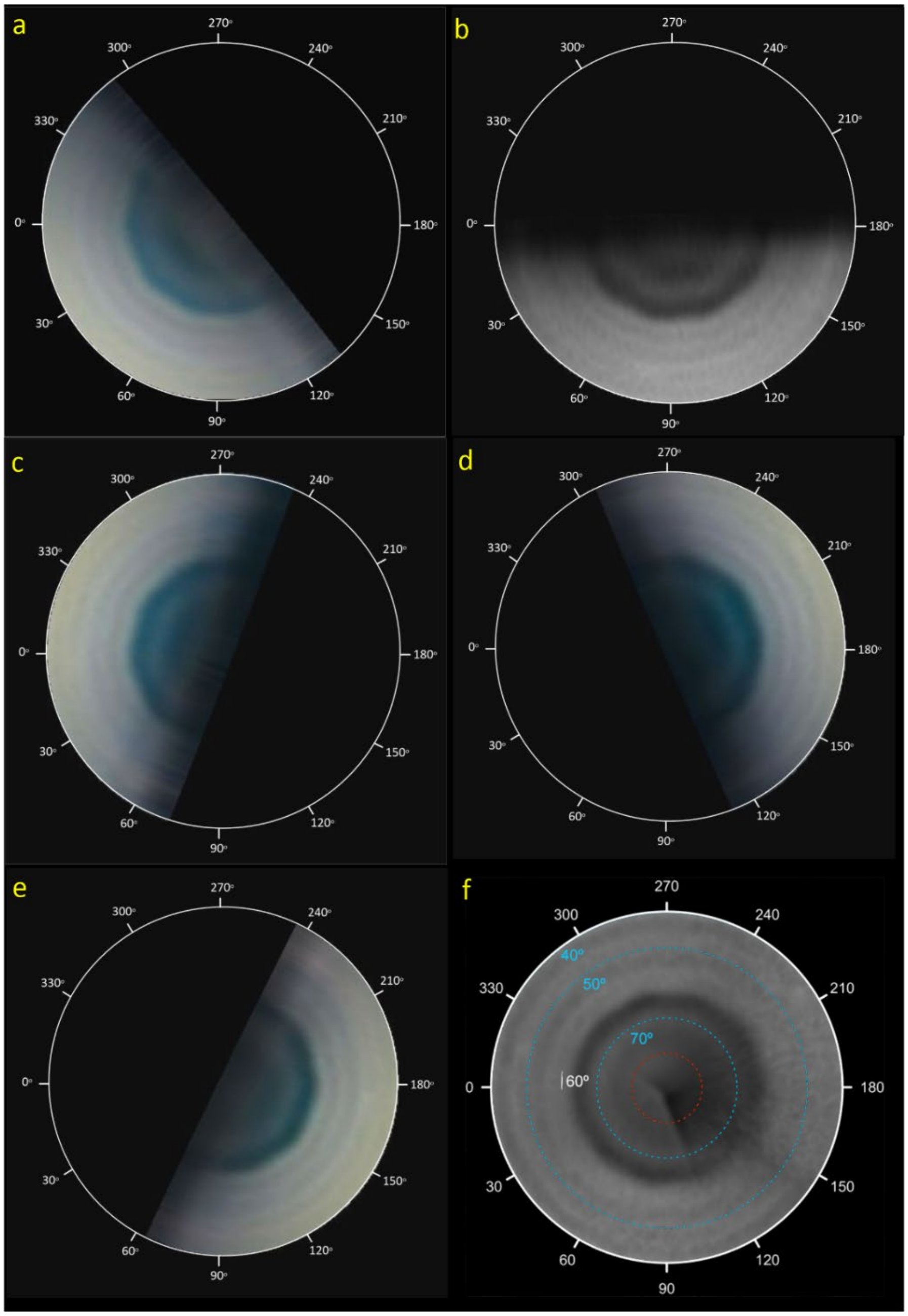

Figure S2. Polar projection maps of Saturn's south hemisphere from 40°S to 90°S from ground-based images obtained in 2025. (a) 26 July, (b) 16 August, (c) 22 August, (d) 25 August, (e) 6 September; (f) 31 August. Maps (a)-(e) from J. P. Oger, map (f) from PlanetCam observations at Calar Alto observatory over 29-31 August. Panels (a),(b) (c),(d),(e) are compositions from images taken with a colour camera and a broadband red filter (transmission wavelength range ~ 642 nm – 1μm). Panel (f) is from images taken with PlanetCam filter I (transmission wavelength range ~ 700 nm – 1μm, (*48*)).

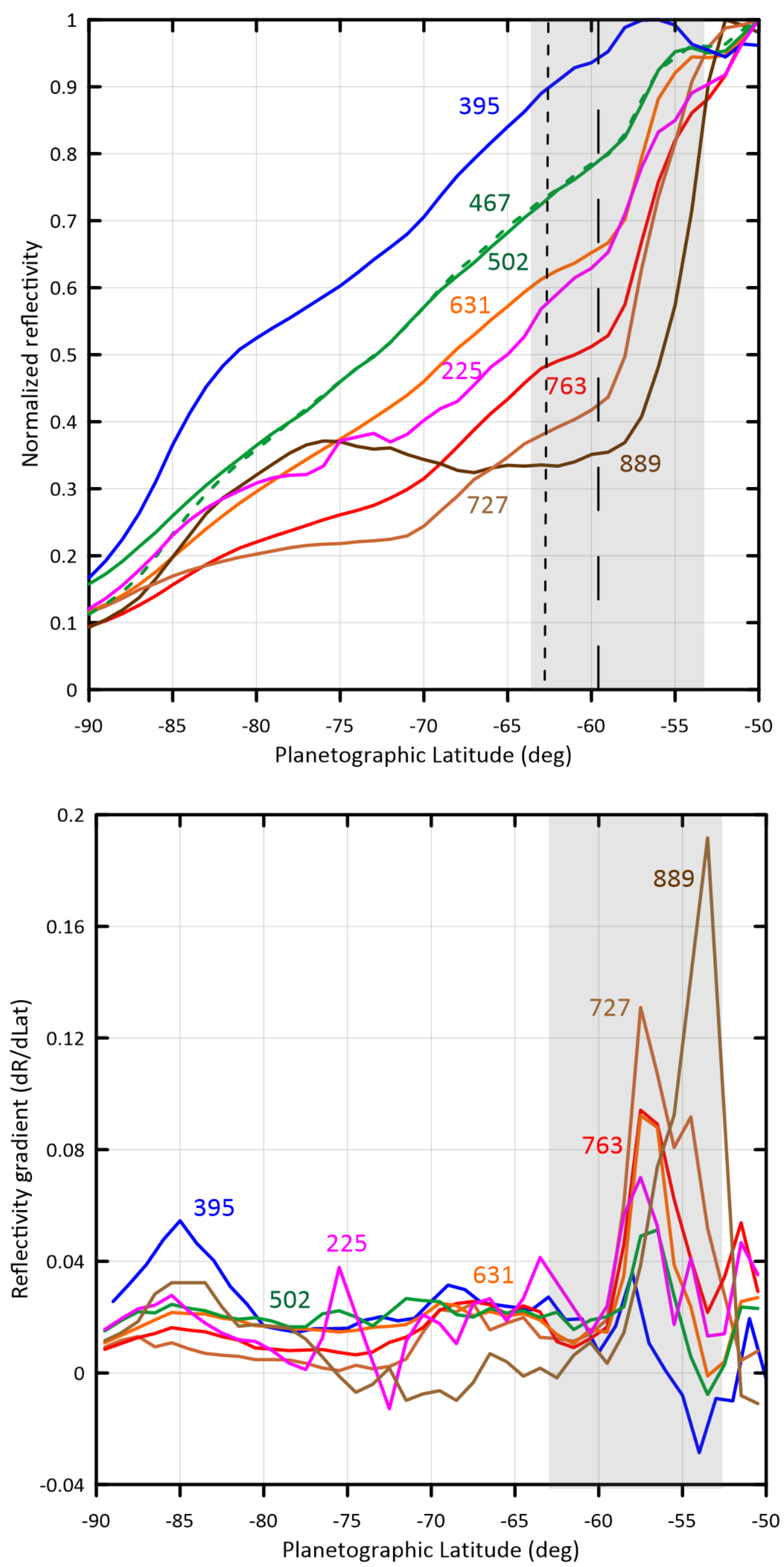

Figure S3. Spectral reflectivity. Upper plot: Reflectivity scans normalized to the maximum for each indicated wavelength. Vertical lines mark the decagon vertices position (dotted line at 763 nm; dashed line at 889 nm). Lower plot: Reflectivity gradient profiles as a function of wavelength. The latitude range occupied by decagonal features is marked by a grey shadow. HST images from 29 August 2025. Details about HST filters are available on ref. (*46-47*).

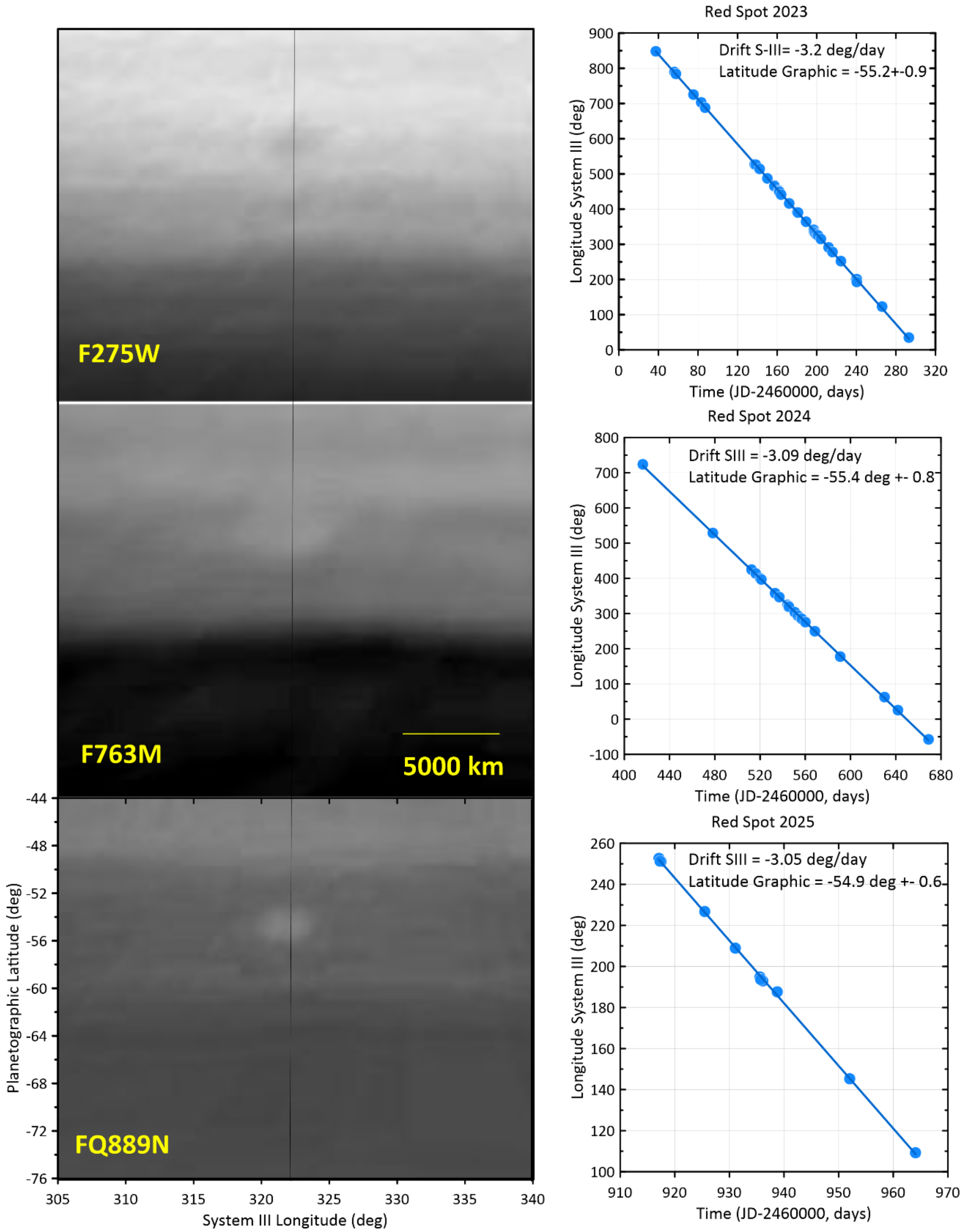

Figure S4. The Red Spot and its motion. At left, HST images of 29 August 2025 in different wavelengths of the anticyclone Red Spot (filters indicated for wavelengths from top to bottom: ultraviolet, red and methane band). At right, drift in System III Longitude of the Red Spot for the years 2023, 2024, 2025 (from top to bottom).

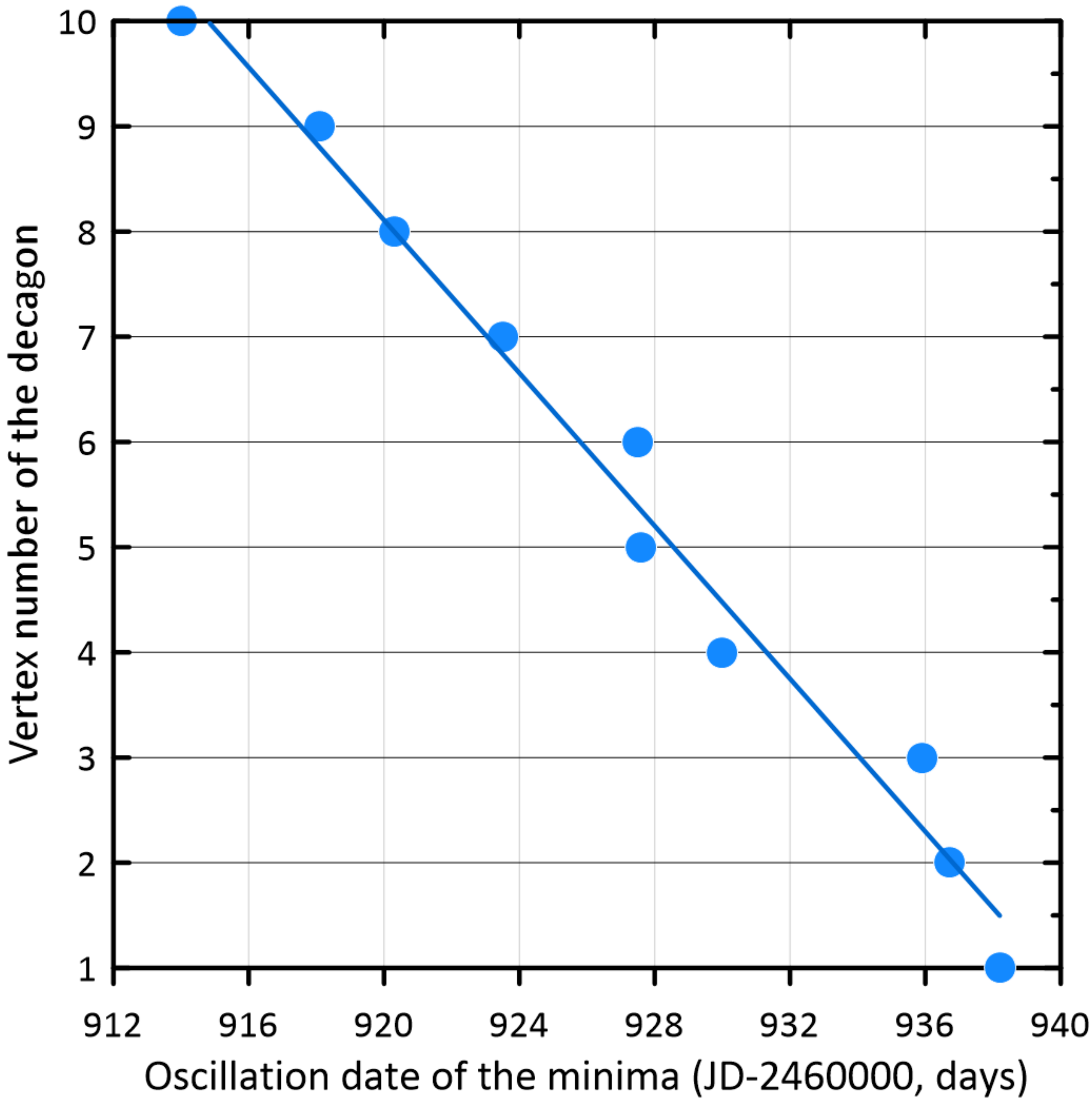

Figure S5. Phase shift of the longitude oscillation for the decagon vertices. The vertices are identified by their number (y-axis) during the observing period (x-axis).

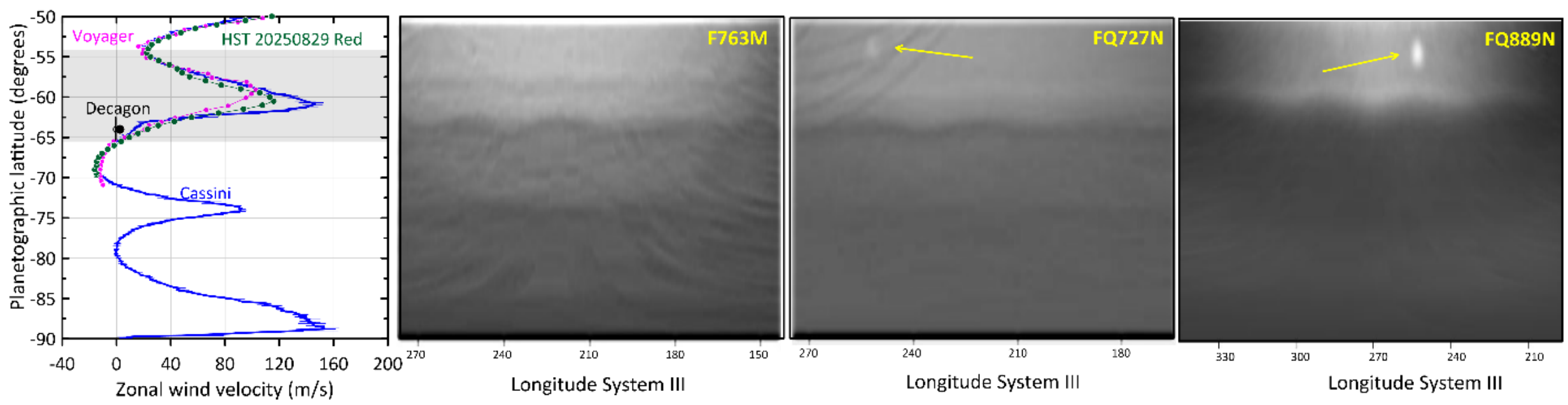

Figure S6. Polar winds in Saturn's Southern Hemisphere and Decagon maps. Polar wind jets in Saturn's Southern Hemisphere in time (left panel) and rectangular projected maps of the Decagon wave from HST images at different wavelengths (as indicated by the filter identification). The wind profiles are from images obtained by the following spacecraft: Voyager 1 and 2 in 1981 (magenta, *13*), Cassini in 2004-2009 (blue, *14-15*) and HST 2025 (green, this work). The decagon velocity is indicated by a black dot and vertical black bar. The meridional extent occupied by the decagon waves is marked by the grey shadow over the wind profile. The yellow arrow marks the Red Spot (RS).

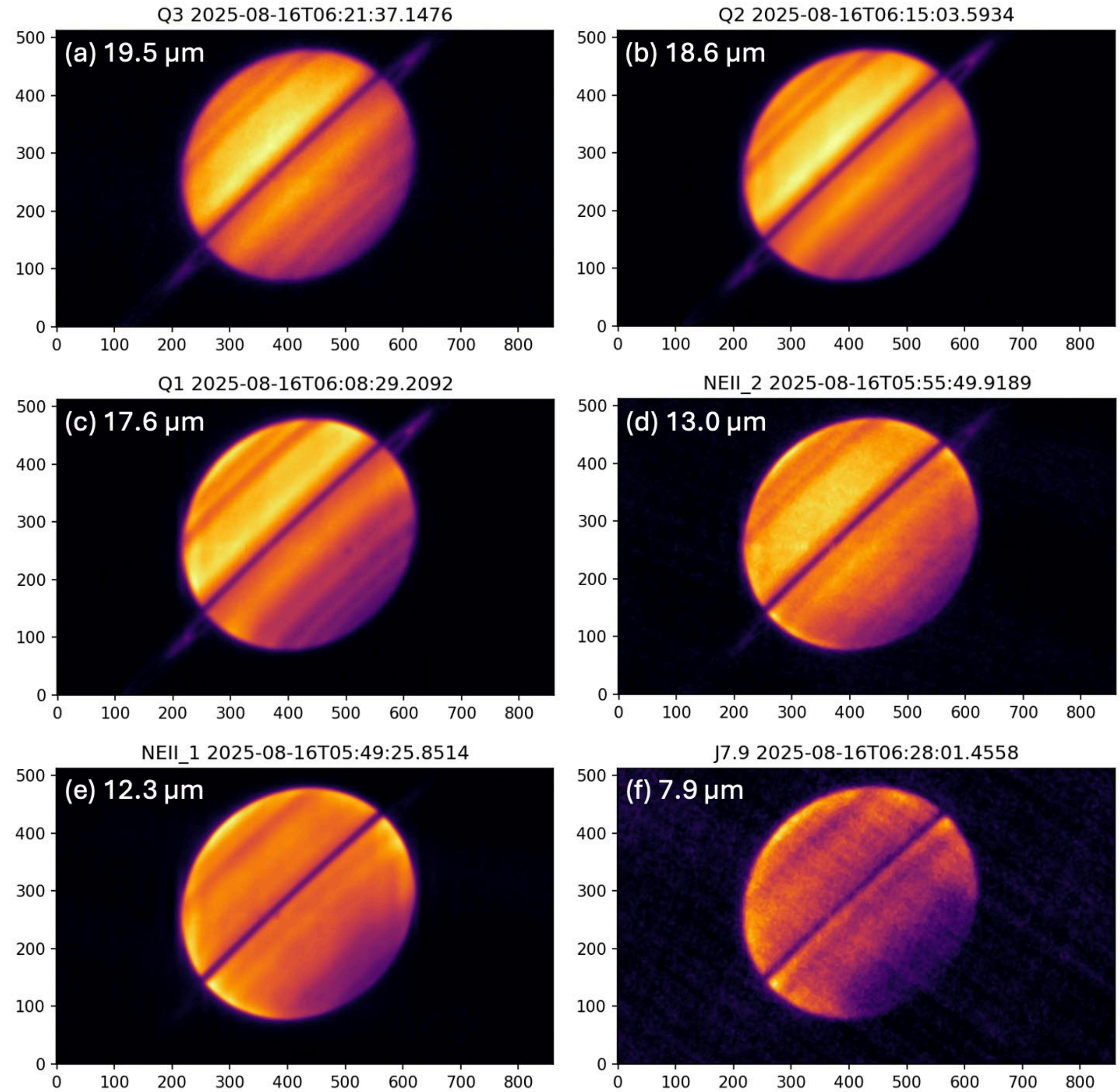

Figure S7. **Infrared images of Saturn.** Montage of VLT/VISIR thermal-IR images of Saturn acquired on 2025-08-16. Data were collected in seven wavelengths, but an image at 8.6 µm is omitted due to low signal-to-noise. Wavelengths between 17.6-19.5 µm sound temperatures in the upper troposphere (100-300 mbar) and reveal belt/zone structure superimposed onto a seasonal gradient (from warm northern autumn, to cool southern spring). Wavelengths at 7.9, 12.3 and 13.0 µm sound stratospheric methane, ethane, and acetylene respectively, and can be used to provide a crude estimate of stratospheric temperatures.

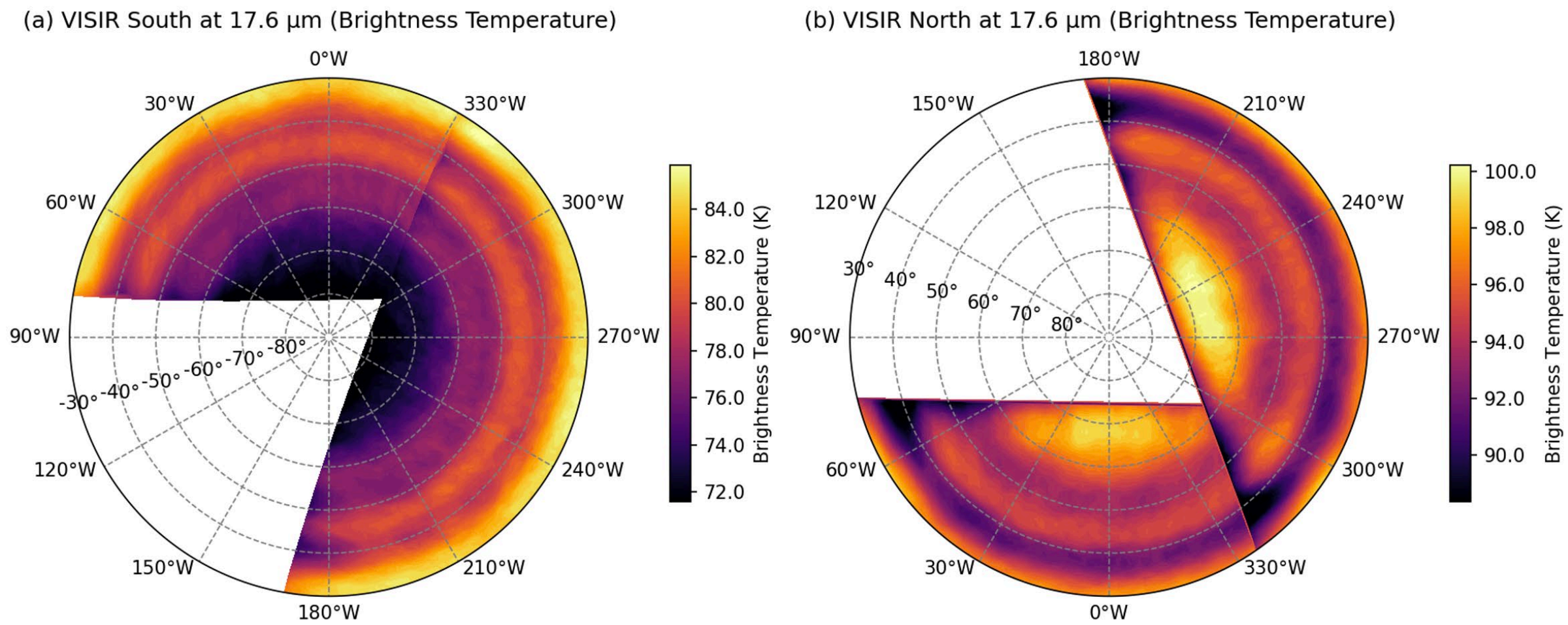

Figure S8. Polar maps in the thermal infrared. Polar projections of VLT/VISIR images of Saturn at 17.6 µm between 30° and 90º in each hemisphere, combining data from 2026-08-16 and 2025-08-17 to map ~270º of longitude. Note the difference in brightness temperature scales due to seasonal differences between the northern and southern hemispheres. Limb-darkening was removed by fitting a polynomial to the centre-to-limb brightness in the equatorial region (omitting ring-obscured latitudes), and then dividing all pixels by this limb correction. The low temperatures of the springtime pole (i.e., low SNR), combined with the high-emission-angle view and low contrast in the banded structure, makes it challenging to see the decagon wave with VISIR. In fact, the north polar hexagon was visible in northern summer in 2017-2020 (*29*), but cannot be seen in 2025.

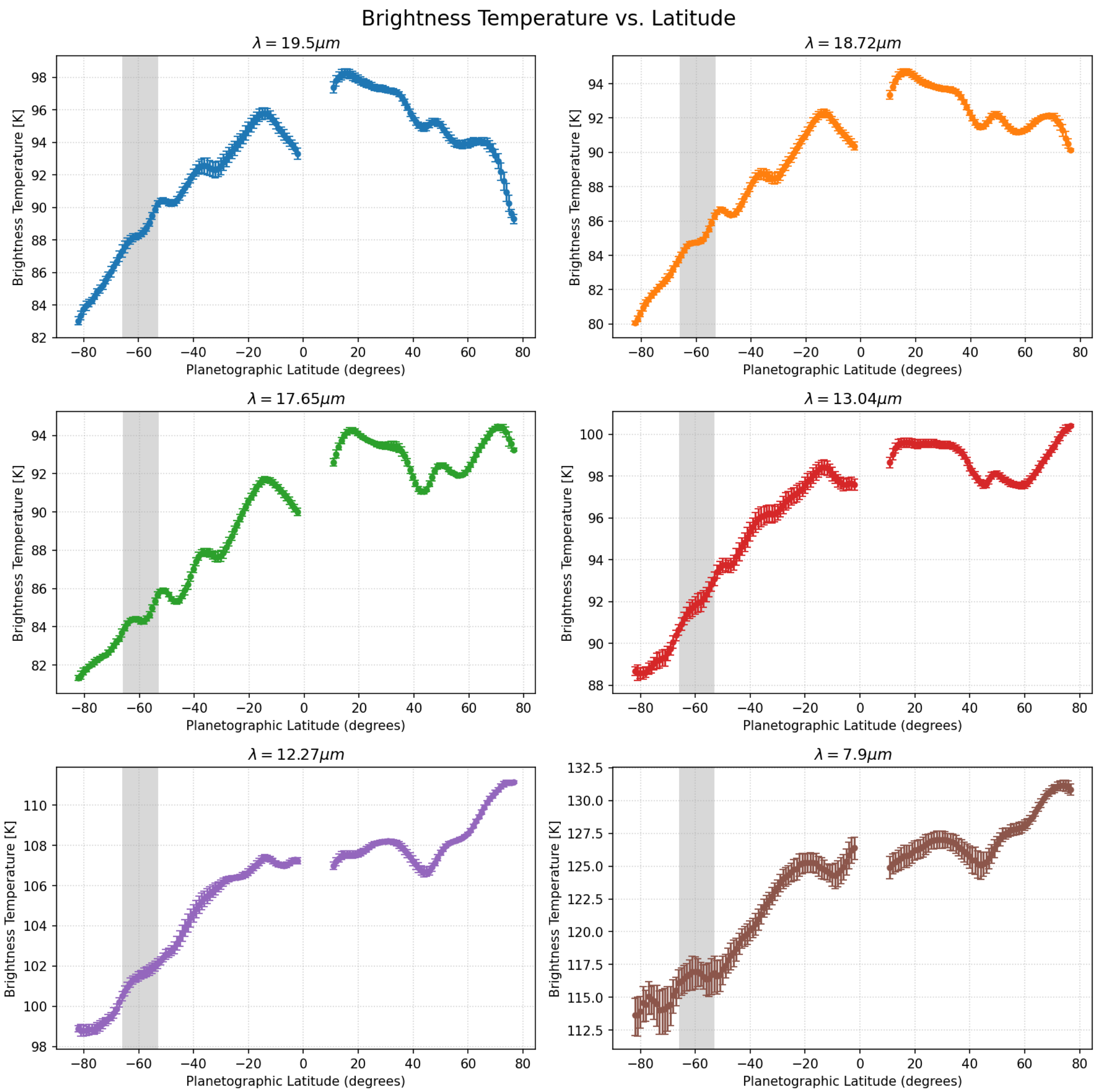

Figure S9. Brightness Temperature meridional scans. North-south zonally averaged brightness temperatures extracted from VISIR images on 2025-08-16. We averaged brightness within ±30º of the central meridian, and make no correction for the observing geometry (i.e., emission angle as a function of latitude). Latitudes between 0-15ºN are omitted due to obscuration by Saturn's rings. The grey band marks the location of the decagon wave (53°S-66ºS). These data were subsequently used to retrieve the zonal-mean temperature as a function of latitude in the main text (Fig. 4).

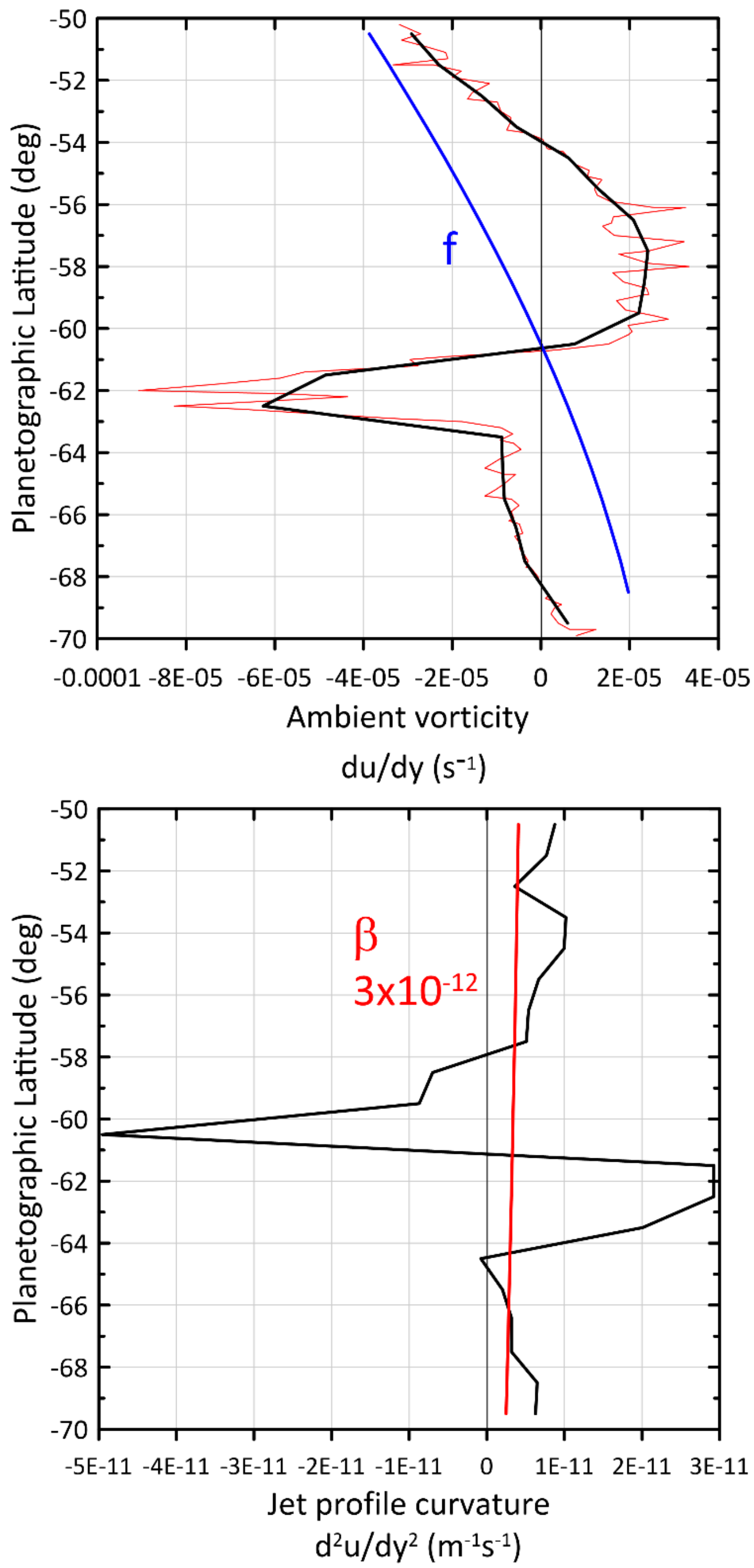

Figure S10. Zonal jet ambient vorticity and curvature profiles. Properties of the zonal jet with peak velocity at latitude 60.7° from Cassini (*15*). Upper panel shows the meridional wind shear (ambient vorticity, red line) and its average in 1° (black line). The blue line shows the parameter βy. The lower panel shows the jet profile curvature (black curve) and the β-parameter.

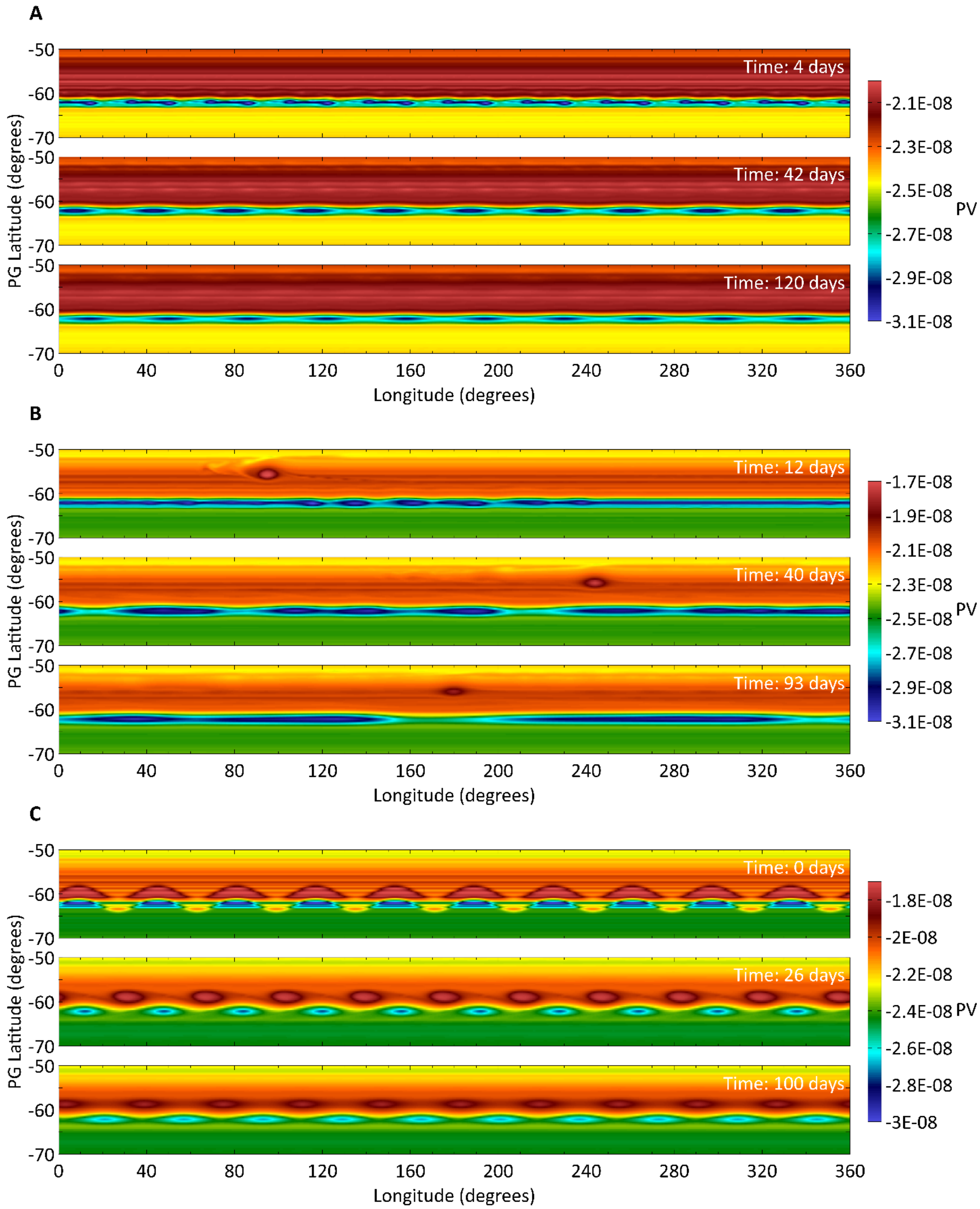

Figure S11. Shallow Water model results. (A) Map showing the evolution of the potential vorticity field (*PV*) generated by an initial periodic disturbance with wavenumber 10 centered on the jet peak at 60.5°S. (B) Map showing the evolution of the *PV* perturbation caused by the presence of the RS anticyclone at 55°S with velocity at periphery V_t = 125 ms^{-1}. (C) Map of the evolution of the *PV* when a periodic sinusoidal wave with amplitude 1.3° and wavenumber 10 initially introduced on the jet peak. For all the cases we use H = 12.5 km, L_D = 1329 km. Polar projection maps of these simulations are presented in main text as Figure 5.

Height sensitivity to wavelength

We present the approximate range of altitudes to which the HST filters showing the decagon are sensitive. Since all of them are in the red wavelength range, the contribution to reflectivity from Rayleigh scattering is negligible. Reflectivity is controlled by methane absorption and by the aerosols present in stratospheric and tropospheric hazes, and in the upper ammonia cloud. We rely on published reference models of clouds and aerosols for Saturn's polar atmosphere (*18-19*).

Table S1. Standard cloud model for Saturn North Polar Region

Layer (acronym)	Pressure range	Aerosol optical depth
Stratospheric haze (SH)	0.001 - 0.1 bar	0.01-0.03
Tropospheric haze (TH)	0.1 – 0.6	1 - 3
Upper cloud (C)	1 - 3	10 - 15

Table S2. Probed altitude ranges by HST filters showing the decagon

Filter	λ_{eff} (nm)	BW_{eff} (nm)	P_{CH4} (τ =1) (bar)	Probed altitude range (bar)	Layer
FQ889N	889.4	9.8	0.057	0.01 – 0.150	SH-TH (upper)
F727N	727.5	6.4	0.57	0.5 – 1.5	TH - C
F763M	760.3	73.9	> 10	1 – 3 (and deeper)	TH - C
F631N	630.4	5.8	> 10	1 – 3 (and deeper)	TH - C
FQ937N	937.2	9.4	> 10	1 – 3 (and deeper)	TH - C

Notes: λ_{eff} (filter effective wavelength), BW_{eff} (filter bandwidth), P_{CH4} (τ =1) (pressure level where methane absorption reaches optical depth = 1), given by

$$P_{CH_4}(\tau = 1) = \frac{<\mu> g}{<k_{eff}> x_V V_0}$$

$<\mu>$ = 2.2 g mol^{-1}, g = 10.6 ms^{-2}, V_0 = 22.4 l, x_V = 4.5x10^{-3}, $<k_{eff}>$ (cm-amagat)$^{-1}$ = 4x10^{-4} (889N nm), 4x10^{-5} (727N), 2x10^{-6} (763M), 4x10^{-7} (937N).

Table S3. Decagon Vertices Movement Measurements

Vertex	System III Longitude	System III Drift	Oscillation Period	Oscillation Amplitude (max)	Date (time) Oscillation minimum	R^2 Oscillation
V1	349.7°	-0.43°/d	31 days	4.9° (12°)	938.2	0.39
V2	313°	-0.47°/d	36 days	6.1° (10°)	936.7	0.74
V3	274.2°	-0.45°/d	32 days	5.7° (12°)	935.9	0.57
V4	244.1°	-0.53°/d	43 days	3.6° (6°)	930	0.38
V5	206.7°	-0.50°/d	34 days	4.8° (9°)	927.6	0.50
V6	170.1°	-0.48°/d	30 days	4.0° (6°)	927.5	0.55
V7	134.5°	-0.42°/d	30 days	3.8° (6°)	923.5	0.61
V8	99.1°	-0.50°/d	29 days	5.3° (8°)	920.3	0.64
V9	62.9°	-0.47°/d	29 days	3.5° (8°)	918.1	0.37
V10	27.6°	-0.44°/d	31 days	4.9° (7°)	914	0.51

Column:

(1) Vertex identification (see Figure 2)
(2) System III Longitude at time 940 (see Figure 2)
(3) Drift from a linear fit (see Figure 2), in degrees/day
(4) Oscillation period from sinusoidal fit
(5) Oscillation amplitude from sinusoidal fit (and Amplitude maximum)
(6) Date (time) of the minimum in the oscillation (see Figure 2)
(7) R^2-oscillation fit; best fit to a sinusoidal function

Range of Parameters used in the Shallow Water model

Tables S4–S6 present the values of the numerical and physical parameters used in the shallow-water (SW) simulations (some of them shown in Figures 5 and S11).

Table S4. Numerical parameters

Parameter	Value
Longitude domain	0° - 360°
Latitude domain	50°S - 70°S
Resolution Latitude	0.2°
Resolution Longitude	0.25°
Temporal resolution (time step)	2 seconds
Time duration	100-125 days

Table S5. Physical parameters

Cases studied in Figure 5	H (km)	λ_0 (°), φ_0 (°)	a (°) , b (°)	n	V_t (ms^{-1})
1- 10 Periodic disturbances	12.5	36m, 61S<br>m=1,2…9	1.4, 1.4	2	25
2- Anticyclone RS	12.5	36, 55S	2.8, 2	2	100
3- Imposed wave (wavenumber 10)	12.5	φ_0 = 61S	-	2	-

Table S6. Range of physical values tested

Cases studied in Figure 5	H (km)	λ_0 (°), φ_0 (°)	a (°) , b (°)	n	V_t (ms^{-1})
1- 10 Periodic disturbances	1-50	36m, 60S-61S-62S<br>m=1,2…9	a = 1.4-2.1<br>b = 1.4-2.1	2	10-25-50-60
2- Anticyclone RS	1-30	36, 54S-55S-56S	a = 2.8-3.1-6.2<br>b = 2.2-3.8	2	25-50-75<br>100-125
3- Imposed wave (wavenumber 10)	1-30	φ_0 = 60S-61S-62S	-	2	-

REFERENCES

1. A. D. Del Genio, R. K. Achterberg, K. H. Baines, F. M. Flasar, P.L. Read, A. Sánchez-Lavega, A. P. Showman, "Saturn atmospheric structure and dynamics" in *Saturn after Cassini-Huygens*, M. Dougherty, L. Esposito, T. Krimigis, Eds. (Springer-Verlag, 2009), pp. 113–159.

2. K. M. Sayanagi, K. H. Baines, U. Dyudina, L. N. Fletcher, A. Sánchez-Lavega, R. A. West, "Saturn's polar atmosphere" in *Saturn in the 21st Century*, K. Baines, F. Flasar, N. Krupp, T. Stallard, Eds. (Cambridge University Press, Cambridge, 2018), pp. 337–376.

3. D. A. Godfrey, A hexagonal feature around Saturn's north pole. *Icarus* **76**, 335–356 (1988).

4. A. Sánchez Lavega, J. Lecacheux, F. Colas, P. Laques, Ground-based observations of Saturn's North Polar Spot and Hexagon. *Science* **260**, 329–332 (1993).

5. K. H. Baines, T. W. Momary, L. N. Fletcher, A. P. Showman, M. Roos-Serote, R. H. Brown, B. J. Buratti, R. N. Clark, P. D. Nicholson, Saturn's north polar cyclone and hexagon at depth revealed by Cassini/VIMS. *Planet. Space Sci.* **57**, 1671–1681 (2009).

6. A. Sánchez-Lavega, T. del Río-Gaztelurrutia, R. Hueso, S. Pérez-Hoyos, E. García-Melendo, A. Antuñano, I. Mendikoa, J. F. Rojas, J. Lillo, D. Barrado-Navascués, J. M. Gomez-Forrellad, C. Go, D. Peach, T. Barry, D. P. Milika, P. Nicholas, A. Wesley, The long-term steady motion of Saturn's hexagon and the stability of its enclosed jet stream under seasonal changes. *Geophys. Res. Lett.* **41**, 1425–1431 (2014).

7. A. Antuñano, T. del Rio-Gaztelurrutia, A. Sánchez-Lavega, R. Hueso, Dynamics of Saturn's polar regions. *J. Geophys. Res. Planets* **120**, 155–176 (2015).

8. L. N. Fletcher, P. G. J. Irwin, G. S. Orton, N. A. Teanby, R. K. Achterberg, G. L. Bjoraker, P. L. Read, A. A. Simon-Miller, C. Howett, R. de Kok, N. Bowles, S. B. Calcutt, B. Hesman, F. M. Flasar, Temperature and composition of Saturn's polar hot spots and hexagon. *Science* **319**, 79–81 (2008).

9. L. N. Fletcher, G. S. Orton, J. A. Sinclair, S. Guerlet, P. L. Read, A. Antuñano, R. K. Achterberg, F. M. Flasar, P. G. J. Irwin, G. L. Bjoraker, J. Hurley, B. E. Hesman, M. Segura, N. Gorius, A. Mamoutkine, S. B. Calcutt, A hexagon in Saturn's northern stratosphere surrounding the emerging summertime polar vortex. *Nat. Commun.* **9**, 3564 (2018).

10. A. Sánchez-Lavega, E. García-Melendo, J. Legarreta, R. Hueso, T. del Río-Gaztelurrutia, J. F. Sanz-Requena, S. Pérez-Hoyos, A. A. Simon, M. H. Wong, M. Soria, J. M. Gómez-Forrellad, T. Barry, M. Delcroix, K. M. Sayanagi, J. J. Blalock, J. L. Gunnarson, U. Dyudina, S. Ewald, A complex storm system and a planetary-scale disturbance in Saturn's north polar atmosphere in 2018. *Nat. Astron.* **4**, 180–187 (2020).

11. R. Hueso, A. Sánchez-Lavega, J. F. Rojas, A. Simon, T. Barry, T. de Rio-Gaztelurrutia, K. M. Sayanagi, A. Antuñano, J. Blalock, F. Colas, M. Delcroix, J. M. Gomez-Forrellad, J. Gunnarson, D. Peach, S. Perez-Hoyos, M. H. Wong, Saturn atmospheric dynamics one year after Cassini: Long-lived features and hexagon's drift rate variations. *Icarus* **336**, 113429 (2020).

12. A. Sánchez-Lavega, E. García-Melendo, T. del Río-Gaztelurrutia, R. Hueso, A. Simon, M. H. Wong, K. Ahrens-Velàsquez, M. Soria, T. Barry, C. Go, C. Foster, Interaction of Saturn's Hexagon with convective storms. *Geophys. Res. Lett.* **48**, e2021GL092461 (2021).

13. A. Sánchez-Lavega, J. F. Rojas, P. V. Sada, Saturn's zonal winds at cloud level. *Icarus* **147**, 405–420 (2000).

14. C. C. Porco, E. Baker, J. Barbara, K. Beurle, A. Brahic, J. A. Burns, S. Charnoz, N. Cooper, D. D. Dawson, A. D. del Genio, T. Denk, L. Dones, U. Dyudina, M. W. Evans, B. Giese, K. Grazier, P. Helfenstein, A. P. Ingersoll, R. A. Jacobson, T. V. Johnson, A. McEwen, C. D. Murray, G. Neukum, W. M. Owen, J. Perry, T. Roatsch, J. Spitale, S. Squyres, P. Thomas, M. Tiscareno, E. Turtle, A. R. Vasavada, J. Veverka, R. Wagner, R. West, Cassini Imaging Science: Initial results on Saturn's atmosphere. *Science* **307**, 1243–1247 (2005).

15. E. García-Melendo, S. Pérez-Hoyos, A. Sánchez-Lavega, R. Hueso, Saturn's zonal wind profile in 2004–2009 from Cassini ISS images and its long-term variability. *Icarus* **215**, 62–74 (2011).

16. A. Sánchez-Lavega, S. Pérez-Hoyos, J. R. Acarreta, R. French, No hexagonal wave around Saturn's southern pole. *Icarus* **160**, 216–219 (2002).

17. A. R. Vasavada, S. M. Hörst, M. R. Kennedy, A. P. Ingersoll, C. C. Porco, A. D. del Genio, R. A. West, Cassini imaging of Saturn: Southern hemisphere winds and vortices. *J. Geophys. Res.* **111**, E05004 (2006).

18. A. Sánchez-Lavega, A. Garcia-Munoz, T. del Rio-Gaztelurrutia, S. Perez-Hoyos, J. F. Sanz-Requena, R. Hueso, S. Guerlet, J. Peralta, Multilayer hazes over Saturn's hexagon from Cassini ISS limb images. *Nat. Commun.* **11**, 2281 (2020).

19. J. F. Sanz-Requena, S. Pérez-Hoyos, A. Sánchez-Lavega, A. Antuñano, P. G. J. Irwin, Haze and cloud structure of Saturn's North Pole and Hexagon Wave from Cassini/ISS imaging. *Icarus* **305**, 284–300 (2018).

20. B. A. Smith, L. Soderblom, R. Batson, P. Bridges, J. Inge, H. Masursky, E. Shoemaker, R. Beebe, J. Boyce, G. Briggs, A. Bunker, S. A. Collins, C. J. Hansen, T. V. Johnson, J. L. Mitchell, R. J. Terrile, A. F. Cook II, J. Cuzzi, J. B. Pollack, G. E. Danielson, A. P. Ingersoll, M. E. Davies, G. E. Hunt, D. Morrison, T. Owen, C. Sagan, J. Veverka, R. Strom, V. E. Suomi, A new look at the saturn system: The Voyager 2 images. *Science* **215**, 504–537 (1982).

21. A. Sánchez-Lavega, J. F. Rojas, J. R. Acarreta, J. Lecacheux, F. Colas, P. V. Sada, New observations and studies of Saturn's long-lived North Polar Spot. *Icarus* **128**, 322–334 (1997).

22. M. Allison, D. A. Godfrey, R. F. Beebe, A wave dynamical interpretation of Saturn's polar hexagon. *Science* **247**, 1061–1063 (1990).

23. R. Hueso, J. Juaristi, J. Legarreta, A. Sánchez-Lavega, J. F. Rojas, S. Erard, B. Cecconi, P. Le Sidaner, The Planetary Virtual Observatory and Laboratory (PVOL) and its integration into the Virtual European Solar and Planetary Access (VESPA). *Planet. Space Sci.* **150**, 22–35 (2018).

24. B. A. Archinal, C. H. Acton, M. F. A'Hearn, A. Conrad, G. J. Consolmagno, T. Duxbury, D. Hestroffer, J. L. Hilton, R. L. Kirk, S. A. Klioner, D. McCarthy, K. Meech, J. Oberst, J. Ping, P. K. Seidelmann, D. J. Tholen, P. C. Thomas, I. P. Williams, Report of the IAU working group on cartographic coordinates and rotational elements: 2015. *Celest. Mech. Dyn. Astron.* **130**, 22 (2018).

25. H. G. Solberg, A 3-month oscillation in the longitude of Jupiter's Red Spot. *Planet. Space Sci.* **17**, 1573–1580 (1969).

26. J. M. Trigo-Rodríguez, A. Sánchez-Lavega, J. M. Gómez, J. Lecacheux, F. Colas, I. Miyazaki, The 90-day oscillations of Jupiter's Great Red Spot revisited. *Planet. Space Sci.* **48**, 331–339 (2000).

27. A. A. Simon, M. H. Wong, P. S. Marcus, P. G. J. Irwin, A detailed study of Jupiter's Great Red Spot over a 90-day oscillation cycle. *Planet. Sci. J.* **5**, 223 (2024).

28. P. Lagage, J. Pel, M. Authier, J. Belorgey, A. Claret, C. Doucet, D. Dubreuil, G. Durand, E. Elswijk, P. Girardot, H. Kaufl, G. Kroes, M. Lortholary, Y. Lussignol, M. Marchesi, E. Pantin, R. Peletier, J. Pirard, J. Pragt, Y. Rio, T. Schoenmaker, R. Siebenmorgen, A. Silber, A. Smette, M. Sterzik, C. Veyssière, "Successful Commissioning of VISIR: The Mid-Infrared VLT instrument," The Messenger (2004), no. 117, pp. 12–16; www.eso.org/sci/publications/messenger/archive/no.117-sep04/messenger-no117-12-16.pdf.

29. J. S. D. Blake, L. N. Fletcher, G. S. Orton, A. Antuñano, M. T. Roman, Y. Kasaba, T. Fujiyoshi, H. Melin, D. Bardet, J. A. Sinclair, M. Es-Sayeh, Saturn's seasonal variability from four decades of ground-based mid-infrared observations. *Icarus* **392**, 115347 (2023).

30. L. N. Fletcher, G. S. Orton, P. Yanamandra-Fisher, B. Fisher, P. D. Parris, P. G. J. Irwin, Retrievals of atmospheric variables on the gas giants from ground-based mid-infrared imaging. *Icarus* **200**, 154–175 (2009).

31. P. L. Read, B. J. Conrath, L. N. Fletcher, P. J. Gierasch, A. A. Simon-Miller, L. C. Zuchowski, Mapping potential vorticity dynamics on Saturn: Zonal mean circulation from Cassini and Voyager data. *Planet. Space Sci.* **57**, 1682–1698 (2009).

32. A. C. Barbosa Aguiar, P. L. Read, R. D. Wordsworth, T. Salter, Y. Hiro Yamazaki, A laboratory model of Saturn’s north polar hexagon. *Icarus* **206**, 755–763 (2010).

33. R. Morales-Juberías, K. M. Sayanagi, A. A. Simon, L. N. Fletcher, R. G. Cosentino, Meandering shallow atmospheric jet as a model of Saturn’s north-polar hexagon. *Astrophys. J. Lett.* **806**, L18 (2015).

34. M. Rostami, V. Zeitlin, A. Spiga, On the dynamical nature of Saturn’s north polar hexagon. *Icarus* **297**, 59–70 (2017).

35. A. Antuñano, T. del Río-Gaztelurrutia, A. Sánchez-Lavega, J. Rodriguez Aseguinolaza, Cloud morphology and dynamics in Saturn's Northern Polar Region. *Icarus* **299**, 117–132 (2018).

36. A. Antuñano, T. del Río-Gaztelurrutia, A. Sánchez-Lavega, P. L. Read, L. N. Fletcher, Potential vorticity of Saturn’s polar regions. *J. Geophys. Res.* **124**, 186–201 (2019).

37. E. García-Melendo, A. Sánchez-Lavega, Shallow water simulations of Saturn's Giant Storms at different latitudes. *Icarus* **286**, 241–260 (2017).

38. A. Sánchez-Lavega, G. Fischer, L. N. Fletcher, E. García-Melendo, B. Hesman, S. Pérez-Hoyos, K. M. Sayanagi, L. A. Sromovsky, “The Great Storm of 2010-2011” in *Saturn in the 21st Century*, K. H. Baines, F. M. Flasar, N. Krupp, T. S. Stallard, Eds. (Cambridge University Press, Cambridge, U.K, 2019), pp. 377–416.

39. R. Morales-Juberías, K. M. Sayanagi, T. E. Dowling, A. P. Ingersoll, Emergence of polar-jet polygons from jet instabilities in a Saturn model. *Icarus* **211**, 1284–1293 (2011).

40. A. Adriani, A. Mura, G. Orton, C. Hansen, F. Altieri, M. L. Moriconi, J. Rogers, G. Eichstädt, T. Momary, A. P. Ingersoll, G. Filacchione, G. Sindoni, F. Tabataba-Vakili, B. M. Dinelli, F. Fabiano, S. J. Bolton, J. E. P. Connerney, S. K. Atreya, J. I. Lunine, F. Tosi, A. Migliorini, D. Grassi, G. Piccioni, R. Noschese, A. Cicchetti, C. Plainaki, A. Olivieri, M. E. O’Neill, D. Turrini, S. Stefani, Clusters of cyclones encircling Jupiter’s poles. *Nature* **555**, 216–219 (2018).

41. R. N. Ibragimov, G. Lin, Longitudinal dimensions of polygon-shaped planetary waves. *J. Appl. Nonlinear Dyn.* **4**, 153–167 (2015).

42. B. F. Farrell, P. J. Ioannou, Statistical state dynamics based theory for the formation and equilibration of Saturn's north polar jet. *Phys. Rev. Fluids* **2**, 073801 (2017).

43. R. K. Yadav, J. Bloxham, Deep rotating convection generates the polar hexagon on Saturn. *Proc. Natl. Acad. Sci. U.S.A.* **117**, 13991–13996 (2020).

44. A. Constantin, R. S. Johnson, Nonlinear atmospheric flow patterns confined to zonal cloud bands: Saturn's hexagon. *J. Atmos. Sci.* **82**, 1815–1832 (2025).

45. A. A. Simon, M. H. Wong, G. S. Orton, First results from the Hubble OPAL program: Jupiter in 2015. *Astrophys. J. Lett.* **812**, 55 (2015).

46. WFC3 Instrument Handbook [accessed 2026]. https://hst-docs.stsci.edu/wfc3ihb/chapter-1-introduction-to-wfc3/1-4-wfc3-quick-reference-guide.

47. Filter data available at Spanish Virtual Observatory, Centro Astrobiología, CSIC [accessed 2026]. https://svo2.cab.inta-csic.es/theory/fps/index.php?&mode=browse&gname=HST&gname2=WFC3_UVIS1.

48. I. Mendikoa, A. Sánchez-Lavega, S. Pérez-Hoyos, R. Hueso, J. F. Rojas, J. López-Santiago, Temporal and spatial variations of Jupiter's and Saturn's absolute reflectivity from 0.38 to 1.7 µm using PlanetCam-UPV/EHU. *Astron. Astrophys.* **607**, A72 (2017).

49. E. García-Melendo, A. Sánchez Lavega, A study of the stability of Jovian zonal winds from HST Images: 1995-2000. *Icarus* **152**, 316–330 (2001).

50. A. Sánchez-Lavega, L. Sromovsky, A. Showman, A. Del Genio, R. Young, R. Hueso, E. García Melendo, Y. Kaspi, G. S. Orton, N. Barrado-Izagirre, D. Choi, J. Barbara, "Gas giants" in *Zonal Jets*, B. Galperin, P. Read, Eds. (Cambridge University Press, 2019), pp. 72–103.

51. O. King, L. N. Fletcher, PlanetMapper: A Python package for visualising, navigating and mapping Solar System observations. *J. Open Source Softw.* **8**, 5728 (2023).

52. P. G. J. Irwin, N. A. Teanby, R. de Kok, L. N. Fletcher, C. J. A. Howett, C. C. C. Tsang, C. F. Wilson, S. B. Calcutt, C. A. Nixon, P. D. Parrish, The NEMESIS planetary atmosphere radiative transfer and retrieval tool. *J. Quant. Spectrosc. Radiat. Transf.* **109**, 1136–1150 (2008).

53. M. Soria, E. Garcia-Melendo, A. Miró, Shallow Worlds 2 v1.2.0, Zenodo (2023), http://doi.org/10.5281/zenodo.10255095.

54. Misulski Archive Space Telescopes, The Hubble Space Telescope Outer Planet Atmospheres Legacy (OPAL) (2026), https://archive.stsci.edu/hlsp/opal.

55. WinJUPOS 12.4.1 (2026), http://jupos.org/gh/download.htm.

56. The Planetary Virtual Observatory Laboratory (PVOL) (2026), http://pvol2.ehu.eus/pvol2/.

57. ALPO Japan, Association of Lunar and Planetary Observers in Japan, Saturn (2026), https://alpo-j.sakura.ne.jp/indexE.htm.
